# Short-term effects of etifoxine on human gut microbiome in healthy men

**DOI:** 10.3389/fnins.2023.1188847

**Published:** 2023-11-14

**Authors:** André Manook, Thomas C. Baghai, Marco Riebel, Caroline Nothdurfter, Jens Volkmar Schwarzbach, André Gessner, Rainer Rupprecht, Andreas Hiergeist

**Affiliations:** ^1^Department of Psychiatry and Psychotherapy, Universität Regensburg, Regensburg, Germany; ^2^Institute of Clinical Microbiology and Hygiene, Universitätsklinikum Regensburg, Regensburg, Germany

**Keywords:** gut microbiome, etifoxine, neurosteroids, GABA-A transmission, Williams design, 16S rRNA amplicon sequencing, *Faecalibacterium duncaniae*, *Roseburia hominis*

## Abstract

**Background:**

Neurosteroids have recently gained in interest as a treatment strategy for affective disorders. Etifoxine is known for its dual mode of action, one of which is to stimulate endogenous neurosteroid synthesis. The gut microbiome has been studied in affective disorders, but it has not been investigated in the context of human etifoxine or neurosteroid interventions.

**Methods:**

We performed a crossover study with 36 healthy male volunteers who received etifoxine versus alprazolam and placebo in a balanced Williams design. Participants were randomized into six sequences and went through three 5-day treatments followed by wash-out phases of 9 days. Bacterial compositions in stool samples were determined by high-throughput 16S rRNA amplicon sequencing.

**Results:**

Gut microbiome analyses revealed no relevant effects between treatments with respect to alpha and beta diversity. Differential abundance analyses yielded etifoxine treatment as the only effect related to changes in microbial features with reductions of *Faecalibacterium duncaniae*, *Roseburia hominis* and *Lactobacillus rogosae* (i.e., *Bacteroides galacturonicus*).

**Conclusion:**

Here we report on the first human investigation of the gut microbiome with short-term etifoxine intervention. Differences in diversity and compositional structure of the microbiome were more likely due to between- subject effects rather than medication. However, five-day treatment with etifoxine reduced the abundance of a few bacterial species. These species are currently seen as beneficial components of a healthy intestinal microbiome. This reduction in abundances may be related to elevated endogenous neurosteroids.

## Introduction

Neurosteroids (Corpéchot et al., 1981; Robel and Baulieu, 1985) have gained in attention for their therapeutic potential in treating affective disorders such as clinical depression or postpartum depression (Gunay and Pinna, 2022). They are physiological members of the steroidome, which are predominantly produced by the nervous system. They are potent positive allosteric modulators at GABA-A receptors (Hosie et al., 2006). One such neurosteroid, allopregnanolone (Paul et al., 2020) and its intravenous and oral formulations, brexanolone and zuranolone, respectively, have recently drawn particular interest for their potential in treating depression (Gunduz-Bruce et al., 2019; Clayton et al., 2023).

Etifoxine is a benzoxazine derivative that has been used to treat anxiety disorders since the 1970s (Boissier et al., 1972; Cottin et al., 2016; European Medical Agency, 2022). Its primary pharmacodynamics are currently regarded as two independent effects on the GABA-A receptor that lead to GABA transmission. It acts directly as a positive allosteric modulator (Schlichter et al., 2000), but affects different subunits than benzodiazepines (Mattei et al., 2019). Additionally, it stimulates endogenous neurosteroid synthesis yielding increased levels of pregnenolone, progesterone and allopregnanolone in the brain (Verleye et al., 2005; do Rego et al., 2015).

Etifoxine is the only clinically approved ligand for the translocator protein 18 kDa (TSPO) thus far (Rupprecht et al., 2022). Its affinity to TSPO is in the micromolar range (Verleye et al., 2005; Costa et al., 2017; Owen et al., 2022), which is around a factor of 1,000 lower than selective TSPO ligands such as PK11195 (Benavides et al., 1983) or XBD173 (Kita et al., 2004; Owen et al., 2011). TSPO has been hypothesized to be a significant mediator in endogenous neurosteroidogenesis (Schlichter et al., 2000). However, independent TSPO-knockout models (Banati et al., 2014; Morohaku et al., 2014) were viable and showed no obvious phenotypic abnormalities. Most importantly, their steroid biosynthesis was nearly unchanged. Moreover, etifoxine can exert its neurosteroidogenic effects independently of TSPO as demonstrated in blocking studies (Verleye et al., 2005; do Rego et al., 2015; Tu et al., 2015) with TSPO ligand PK11195. The corresponding pathways, however, still remain to be elucidated. For future studies, this also involves the study of TSPO expression and function in human tissue samples like brain tissue derived from brain tumors, or during other necessary neurosurgical interventions, or from *post mortem* material.

Alterations in the gut microbiome along the microbiota-gut-brain axis (Dinan and Cryan, 2012) in affective disorders such as depression are common (Diaz Heijtz et al., 2011; Bastiaanssen et al., 2020; Han et al., 2022). They may be caused by a multitude of possible influences including direct interactions via the vagus nerve and the enteric nervous system, overregulation of the hypothalamic–pituitary–adrenal (HPA) axis, proinflammatory immunomodulation and changed behavior including nutrition, diurnal rhythms, sleep and stressful interactions. The search for disease-related enterotypes has persisted over the years (Arumugam et al., 2011; Valles-Colomer et al., 2019). Recent work with two large Belgian and Dutch cohorts (Valles-Colomer et al., 2019) has shown, for example, that a diagnosis of depression corresponded more likely to a certain enterotype including lower bacterial loads and reduced abundance of butyrate-producing genera like *Faecalibacterium*. Moreover, depression-like symptoms could be induced in rodent models by transferring stool from depressed patients (Kelly et al., 2016; Zheng et al., 2016).

Only a few studies have addressed the impact of neurosteroids on the gut microbiome, despite well-known gut-brain connections via the vagus nerve and enteric nervous system (So and Savidge, 2022). One research group has described how neurosteroid levels were affected in the brains of germ-free animals versus conventionally colonized controls (Diviccaro et al., 2021), and conversely how increased levels of pregnenolone and allopregnanolone in a streptozotocin-induced rat model had an impact on the gut microbiome (Diviccaro et al., 2023).

Interactions between benzodiazepines and the gut were being initially investigated long before high-throughput sequencing enabled high-resolution insights into the complex molecular structure of intestinal microbiota. Fujii et al. (1987) incubated diluted fresh feces from healthy subjects with bromazepam and found that about 80% of the bromazepam was degraded in the fresh fecal suspension, while it was not degraded in sterilized feces. Hepatic encephalopathy was a model disease for the discovery of endogenous benzodiazepines. The human body can only produce these endocepines with the help of the gut microbiome that delivers their precursors (Yurdaydin et al., 1995). These observations have made the gut microbiome a dominant regulator of endocepine homoeostasis (Skolnick and Greig, 2019), fostering speculations that gut endocepines may play an important role in the regulatory processes of sleep and wakefulness, as well as their corresponding metabolomics (Thaiss et al., 2014, 2016).

Allowing participants of a clinical study to “cross over” from one treatment to another - in contrast to parallel group designs - has been sound tradition in medical research for more than 80 years (Jones and Kenward, 2014). It enables researchers to look at various interventions within the same subjects, i.e., repeated within-subject measures, provided that each subject returns to their original state in between treatment periods, for example via flush-out interlacing. One drawback to such a design is the potential of “carry-over effects,” which describe that any consequence of prior treatments may still be influential later on during the trial (similar to “confounding” in other designs). Williams (1949) enhanced previously existing crossover designs by balancing first-order carryover effects for any number of treatments. First order, in this case, relates to a previous treatment in a sequence, while second order, for example, would relate to an intervention prior to the previous one.

**Figure 1:**
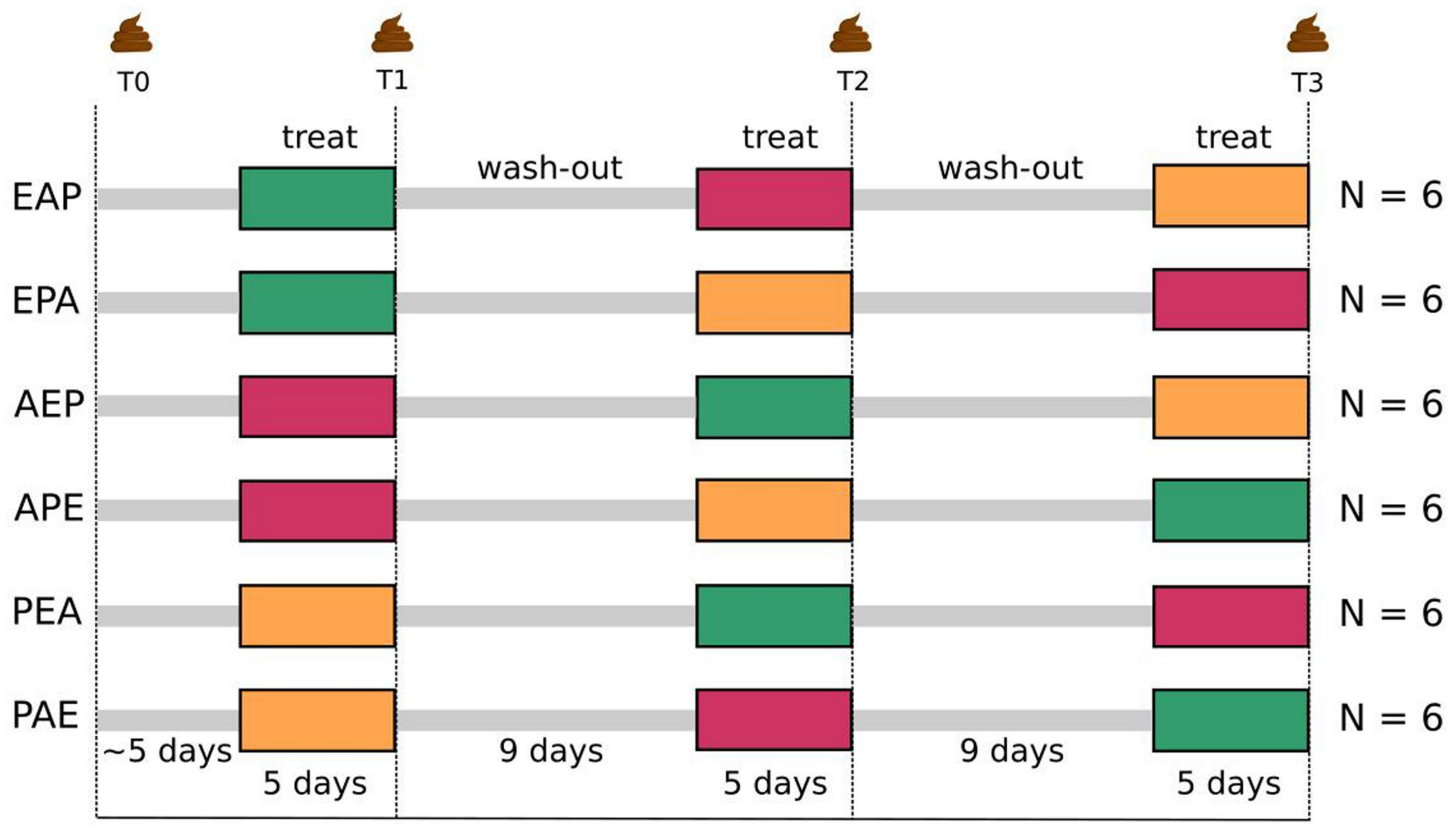
Schematic workflow of balanced Williams crossover design comparing etifoxine (150 mg/d) with alprazolam (1.5 mg/d) and placebo (three “treatments”). Fifty-four men were screened as study prospects for eligibility. Thirty-six of them met inclusion criteria and were randomized into two latin squares of six sequences with six participants each. Three sequential periods are described as timepoints T1, T2, und T3 with an initial baseline measurement (T0) as reference. This Williams design is therefore a 3-treatments, 3-periods and 6-sequences balanced crossover design. Treatments lasted for 5 days followed by 9 days of wash-out. Microbiome was sampled on the last day of treatment. Participants were between 20 and 50 years old (mean = 27.7, SD = 6.9).

Interactions of neurosteroids with the human gut microbiome, including the impact of etifoxine, remain to be elucidated. Consequently, we performed a crossover study using a balanced Williams design (Williams, 1949) with 36 healthy male participants receiving the neurosteroidogenic non-benzodiazepine etifoxine with the benzodiazepine alprazolam and placebo (Figure 1) to investigate the effects of etifoxine on the human gut microbiome.

## Materials and methods

### Study design

The study protocol was designed as a balanced Williams crossover design (Williams, 1949). We decided in favor of using a placebo as one of three possible treatments. Hence, we set up a 3-treatment and 3-period design in which 36 healthy male volunteers were randomized into two latin squares of 6-sequences, such that groups of six participants were treated along the same sequence (Figure 1). In the following, we use the term *time-points* (T1, T2, and T3) interchangeably with the usual design term *periods*.

Each treatment was administered to each participant for 5 days with 9 days of wash-out in between. Consequently, each participant “crossed over” from one treatment to another, but in different sequences that were balanced for first-order carry-over effects. The three treatments were etifoxine (*E*) compared to an established benzodiazepine (alprazolam, *A*) and a placebo (*P*). Applied daily doses of drugs were three times 50 mg of etifoxine (150 mg/day) and three times 0.5 mg of alprazolam (1.5 mg/day). Drugs and placebo were given double-blind as capsules for oral intake with identical appearance. The base composition of the placebo capsule content was identical to capsules that additionally contained drugs.

The definition of an appropriate wash-out period is important since investigating various interventions within the same subjects, i.e., repeated measures, requires that each subject returns to their original state in between treatment periods. Time to reach maximum in blood for etifoxine is 2–3 h, after which it is metabolized rapidly into several metabolites. The longest known half-life of the active metabolites is about 20 h (Choi and Kim, 2015). Consequently, more than 98% of the drug should be eliminated after 6 days and more than 99.8% after nine. Little is known about the kinetics of etifoxine further downstream, and longer wash-out times would take into account the unknown kinetics of biological effects (Owen et al., 2022). However, the study protocol for 36 participants also needed to be feasible, and 2-week blocks (5 plus 9 days) were a sound, reasonable and ethical choice in this regard. The elimination half-life of alprazolam is about 15–16 h and thus shorter than that for etifoxine (Verster and Volkerts, 2004) so that the considerations above also applied to alprazolam.

Initial baseline measurements shortly before the beginning of the trial enabled quality control and building of appropriate analysis measures.

### Human subjects

Healthy male volunteers were recruited at the Department of Psychiatry and Psychotherapy, Universität Regensburg. Only male participants were selected for this study to minimize the influence of hormonal fluctuations. This was of particular relevance for this study since hormones such as progesterone are precursors for neurosteroids like allopregnanolone, and the gut microbiome is known to vary throughout the menstrual cycle.

Exclusion criteria specific to gut microbiome investigations included various nutritional intolerances, celiac disease and non-celiac gluten sensitivity as well as irritable bowel syndrome. Furthermore, the use of antibiotics was not allowed within the last 6 months and changes in diet were not permitted within the last 3 months. Also, more than 3 kg of weight change during the last 3 months was considered an exclusion criterion.

Willingness to abstain from alcohol, driving, operating heavy machinery or engaging in other physically dangerous activities during the pharmaceutical intervention periods were key requirements for study participation. Tolerability of study medication was assessed by the Visual Analogue Scale (Aitken, 1969) and a non-standardized 18-item questionnaire including an open answer section. The Mini International Neuropsychiatric Interview (Sheehan et al., 1998) was used as a screening tool for mental health. Physical health was verified by a physician who, besides controlling for inclusion and exclusion criteria, assessed medical history, conducted a physical examination, and assessed vital signs including ECG and blood work with a particular focus on heart, liver and kidney function. Possible drug usage was ruled out by urine testing.

The trial was conducted at the Department of Psychiatry and Psychotherapy (Universität Regensburg, Regensburg, Germany) from August 2020 to December 2021. It complied with the Declaration of Helsinki and with the Guidelines for Good Clinical Practice of the International Conference on Harmonization as well as with the Arzneimittelgesetz (AMG) in Germany (i.e., Medicinal Products Act). The ethics committee at the Universität Regensburg and the Bundesinstitut für Arzneimittel und Medizinprodukte (BfArM, i.e., Federal Institute for Drugs and Medical Devices) approved the study plan. The clinical trial was registered in the European Clinical Trials Register (EudraCT number: 2018-002181-40) and the German Clinical Trials Register (DRKS-ID: DRKS00020267) as well as with regional authorities. All participants gave written informed consent and were compensated with EUR 1300 if they completed the study.

### Stool sampling and storage

Participants were personally trained by a study nurse with experience in stool sampling to properly deploy and apply a dedicated sterile paper slip for stool collection (Süsse Labortechnik, Gudensberg, Germany) in their toilet bowl at home. The training included the transfer procedure of an appropriate amount of stool from the sterile paper slip into a stool sampling and storage-kit containing a solution to stabilize microbial DNA (MaGix PBI, microBIOMix, Regensburg, Germany), followed by shaking to achieve proper buffer immersion. Participants received the material for use at home including a supplementary photographic manual. Filled storage kits were returned to the study nurse and stored at −80°C. There were no freeze–thaw cycles until the day of extraction. All samples were thawed and preprocessed as a single batch and subsequently analyzed in a single sequencing run.

### 16S rDNA amplicon sequencing and read preprocessing

Microbial DNA was isolated from a volume of stabilization buffer corresponding to 50 mg of original fecal material. Stool suspensions were pre-treated by bead beating on a TissueLyzer II instrument (Qiagen, Hilden, Germany) using Lysing Matrix Y beads (MP Biomedicals, Solon, OH, United States) followed by purification of stool lysates by the MagNA Pure 96 system (Roche Diagnostics, Rotkreuz, Switzerland).

Bacterial 16S rDNA copy numbers were quantified from extracted DNA as previously described (Stammler et al., 2016). In detail, total bacterial 16S rRNA gene copy numbers were determined within the isolated DNA by qPCR on a LightCycler 480 II Instrument (Roche Diagnostics, Rotkreuz, Switzerland). PCR reactions included 1 μM each of universal eubacterial 16S rRNA gene primers 764F and 907R and the LightCycler 480 SYBR Green I Master kit (Roche Diagnostics, Rotkreuz, Switzerland). Quantitative PCRs were performed over 40 cycles (95°C for 10 s, 60°C for 15 s and 72°C for 15 s) with an initial 10 min hot start at 95°C. Complex PCR amplicon mixtures of full length 16S rRNA genes amplified from human fecal DNA were cloned into pGEM TEasy (Invitrogen, Thermo Fisher Scientific, Waltham, MA, United States) and served as a quantification standard.

Microbiome sequencing was conducted according to a DIN EN ISO 15189 accredited workflow. Briefly, the V1-V3 and the V3–V4 variable regions of the 16S rRNA gene were amplified in two separate PCR reactions for each sample using universal primer pairs S-D-Bact-0008-c- S-20/S-D-Bact-0517-a-A-18 and S-D-Bact-0341-b-S-17/S-D-Bact-0785-a-A-21, respectively. Barcoded PCR products of both V-regions and all samples were pooled and purified with AmpureXP Beads (Beckman Coulter, Indianapolis, IN, United States). The sequencing library was quantified with the Ion Library TaqMan™ Quantitation Kit and resulting amplicons were sequenced on an Ion GeneStudio S5 Plus instrument (Thermo Fisher Scientific, Waltham, MA, United States). Raw sequencing data was retrieved from Torrent Suite 5.18 and further subjected to cutadapt 4.1 for adapter and primer removal and demultiplexing, followed by sequence filtering with a quality cutoff of 15 within a sliding window of 10 bases using Trimmomatic 0.39. DNA sequences shorter than 250 bases were removed, and generation of zero-radius operational taxonomic units (zOTUs) and taxonomic classification was performed.

Quality-filtered sequencing data was further processed using a vsearch 2.22.1-based pipeline. Reads with more than five expected errors were removed. Zero-radius OTUs (zOTUs) were built from quality-filtered reads applying an alpha value of 2 and a minimum size of 5 reads. Chimeric sequences were removed using the uchime3_denovo algorithm. Filtered reads with 98 percent pairwise identities were mapped back to non-chimeric zOTUs by applying the usearch_global algorithm. Taxonomy was assigned in R 4.2.2 using the IDTAXA classifier from DECIPHER 2.26.0 together with the All-Species Living Tree database version 06.2022. Here, a 98 percent bootstrap cutoff was used to descend the tree, and taxonomy was reported at each taxonomic level with a confidence value threshold of 40.

### Statistical analyses

#### Model design

The study design described above (Food and Drug Administration (FDA), 2001; Jones and Kenward, 2014) was captured in a full linear mixed model (Baayen et al., 2008; Singmann and Kellen, 2019). Treatments, periods (i.e., time-points), and sequences were used as fixed effects, while participants were considered as random effects as well as an interaction between participants and their randomized sequences. Assumptions for linear regression were tested in advance, including outlier evaluation, normality testing (Shapiro–Wilk) and testing for homogeneity of variances (Levene’s test).

#### R and R packages

All statistical calculations and graph plottings were performed in R (version 4.2.2) within RStudio (2022.12.0, build 353) using RMarkdown. Deployed functions in R and the R packages they originate from were written in the notation <package>::<function> in the corresponding sections.

An overview of the complete study cohort and its sequence groups including age and body mass index (BMI) was calculated and preformatted with table1::table1 (table1, version 1.4.3). Linear mixed effects models were fitted by restricted maximum likelihood (REML) with lmerTest::lmer (lmerTest, version 3.1–3; lme4, version 1.1–31), random slopes and intercepts were set according to the model design described above. Statistical inference was performed using likelihood-ratio tests after nested model reductions (with lmerTest::anova). Post-hoc pairwise testing for individual effects of LME was conducted with emmeans::emmeans (emmeans, version 1.8.4–1).

#### Microbial diversity indices

Bacterial alpha diversity was described in terms of bacterial richness, which was represented by the sum of observed zOTUs (*Observed Species*) as well as the effective number of species (Hill number) for each sample. Furthermore, it was calculated with Inverse Simpson and Shannon indices using the mia package (version 1.1.7) in R. Alpha diversity describes bacterial diversity in a single sample, taking into account the overall number of species present in the sample (richness, *Observed Species*), while indices like *Inverse Simpson* additionally consider their proportional distribution.

Bacterial beta diversity was calculated by Principal Coordinates Analyses (PCoA) using ecodist::pcoa (ecodist, version 2.0.9) after computation of *Bray-Curtis* distances with vegan::vegdist (vegan, version 2.6.4) or *Generalized UniFrac* distances (GUniFrac, version 1.7) with an alpha value of 0.5. Beta diversity is a measure that describes the overall dissimilarity of bacterial communities between at least two samples. Two perspectives on beta diversity were calculated. First, time-series were computed for which beta diversity was restricted to the four samples provided by individual study participants, and second, an overall calculation of beta diversity for all 142 samples of the investigation.

Significance between groups was tested with Permutational Multivariate Analysis of Variance (PERMANOVA) of Bray-Curtis and generalized UniFrac distances with vegan::adonis2 followed by multilevel pairwise comparisons with pairwiseadonis2 (version 0.4). Homogeneity of group dispersions was tested with vegan::betadisper. For this, post-hoc pairwise testing was conducted with stats::TukeyHSD. Additionally, group differences were analyzed by distance-based redundancy analysis (dbRDA) in vegan (vegan::dbrda) followed by pairwise comparisons for group levels with biodiversityR::multiconstrained (biodiversityR, version 2.15-1).

#### Differential abundance analyses

Differential abundances between medication groups were analyzed with two different methods that allowed for deployment of our full LME. For MicrobiomeStat::LinDA (*Linear Model for Differential Abundance Analysis of High-dimensional Compositional Data*; MicrobiomeStat, version 1.1) (Zhou et al., 2022), features below a mean abundance cutoff of 0.1 percent over all samples were filtered. zOTUs below an alpha value of 0.1 were regarded as significantly different. For Maaslin2 (*Multivariable Association Discovery in Population-scale Meta-omics Studies*; Maaslin2, version 1.12.0) (Mallick et al., 2021), the read counts matrix was normalized by total sum scaling followed by log transformation. Features below a mean abundance of 0.1 percent were filtered prior to analysis. Features below the default cutoff of 0.1 for *p*-values adjusted by the Benjamini & Hochberg procedure were regarded as significantly different between compared groups.

## Results

### Study participants

Fifty-four male volunteers were initially screened for eligibility as study prospects, of which 36 met inclusion criteria and were then randomized into one of six sequence groups. Participants were between 20 and 50 years old (mean = 27.7, SD = 6.9). Age and body mass index (BMI) did not differ between the six sequence groups (Table 1), and did not show any significant effects in the subsequent microbiome analyses. Pharmaceutical side effects during treatment periods, as indicated by Visual Analogue Scale measures, previously showed alprazolam to affect both concentration and wakefulness, while etifoxine only affected concentration (Riebel et al., 2023).

**Table 1:** Study cohort characteristics regarding age and body mass index (BMI) of 36 healthy male participants who were randomized into six sequences within a balanced Williams crossover design for the study of etifoxine (*E*) versus alprazolam (*A*) and placebo (*P*).

	AEP (*N* = 6)	APE (*N* = 6)	EAP (*N* = 6)	EPA (*N* = 6)	PAE (*N* = 6)	PEA (*N* = 6)	Overall (*N* = 36)
**Age**							
Mean (SD)	28.1 (4.89)	26.6 (5.18)	30.2 (7.63)	27.7 (8.73)	25.5 (3.92)	28.2 (11.0)	27.7 (6.94)
median [Min, Max]	28.3 [20.0, 34.6]	25.0 [22.8, 36.8]	27.4 [25.3, 45.6]	24.5 [20.4, 44.3]	25.8 [20.2, 30.5]	24.8 [21.6, 50.4]	26.1 [20.0, 50.4]
**BMI**							
Mean (SD)	23.8 (2.81)	25.6 (3.69)	25.1 (2.32)	23.7 (2.72)	24.6 (4.59)	22.6 (1.77)	24.2 (3.05)
Median [min, max]	23.5 [20.7, 28.1]	24.6 [21.6, 31.9]	24.8 [22.1, 28.7]	23.5 [20.6, 27.5]	23.2 [20.0, 32.1]	22.5 [20.7, 25.7]	23.8 [20.0, 32.1]

### Stool preprocessing

One hundred and forty-two stool samples from 36 participants at four time-points (baseline and three study periods) were collected in storage-kits containing a solution to stabilize microbial DNA. Two samples (sequence EAP, periods two and three) from only one single participant could not be collected.

Stool samples were collected in three different batches of prepared storage-kits, ensuring that all samples from one participant were collected with kits from the same batch. Batches covered 13, 12 and 11 participants and did not show any significant differences in bacterial compositions. 16S rDNA copy numbers did not vary significantly between batches, also indicating homogeneity in collected stool suspensions. Frozen storage at −80 degrees Celsius varied between 30 and 93 weeks for baseline samples and had no significant impact on bacterial compositions.

### Test–retest variability

Initial baseline measurements before the beginning of the trial provided the opportunity to evaluate test–retest reliability of microbiome sampling for participants who were randomized into sequence groups that started with placebo (sequences PAE and PEA). Thus, for these twelve participants, there was no pharmacological intervention during the first two microbiome samplings. These were therefore used as test and retest measurements to evaluate the overall variability of both alpha and beta diversity in the context of inter-individual differences.

**Figure 2:**
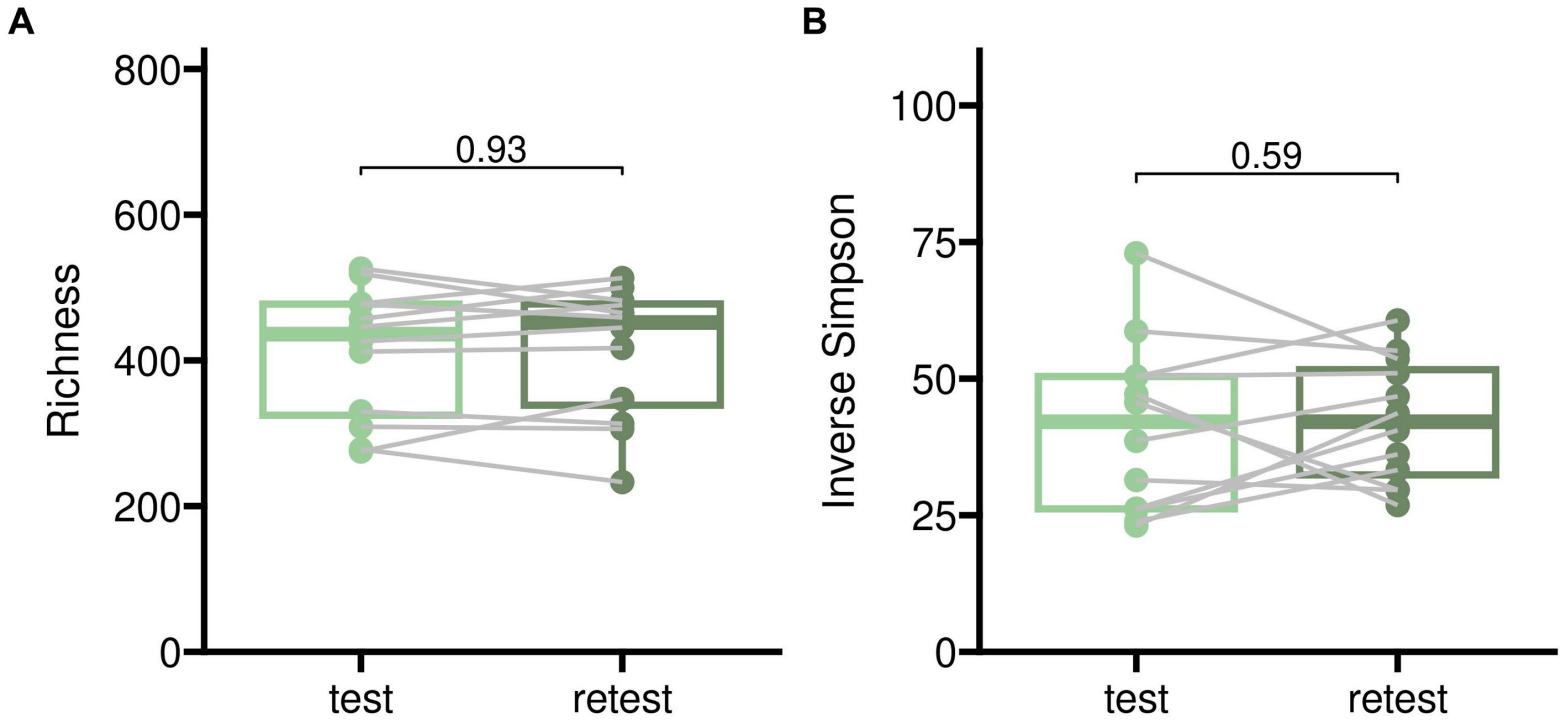
Test–retest analysis for alpha diversity of gut microbiome samples from twelve participants randomized into sequences PAE and PEA. *Test* corresponds to an initial baseline measurement before beginning of the trial and *retest* to sampling after 5 days of the first treatment in case of placebo. The time difference between test and retest was 5 days in median. Two different indices for alpha diversity are shown to account for microbial richness alone (panel **A**) and proportional abundance (*Inverse Simpson*, panel **B**).

**Figure 3:**
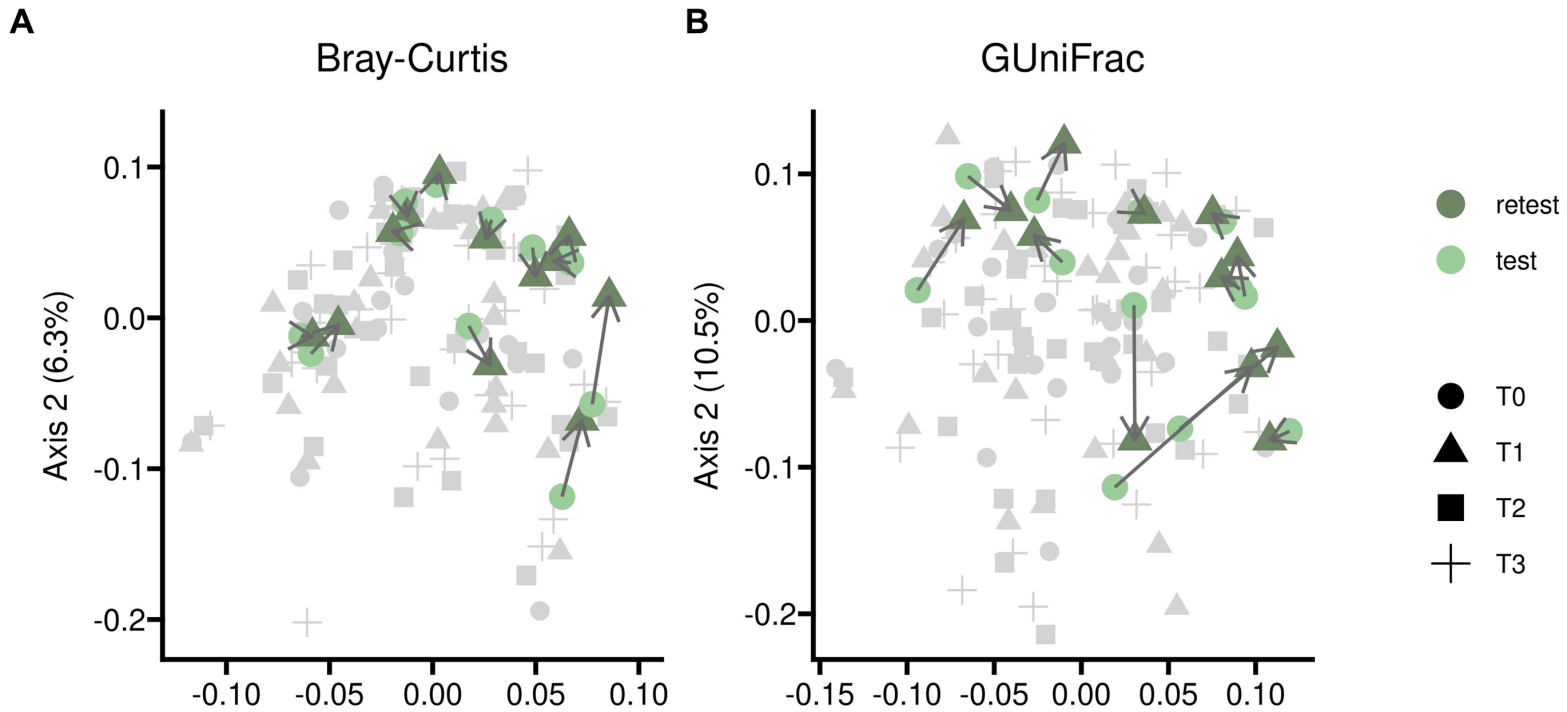
Test–retest analysis for beta diversity of gut microbiome samples from twelve participants randomized into sequences PAE and PEA. Samples were taken at an initial baseline measurement shortly before the trial began (*T0*) and after 5 days of the first treatment (*T1*) in case of placebo. Time difference between test and retest was 5 days in median. Beta diversity was assessed by Principal Coordinates analysis based on two different measures, *Bray-Curtis* (panel **A**) and *Generalized UniFrac* (panel **B**) distances; the latter additionally implements phylogenetic information. The course of individual participants is indicated with connecting arrows. The complete dataset is shown in light gray for better orientation on overall variability of the data.

Test–retests for alpha diversity as described in Figure 2 showed only minor disparities when comparing baseline measurements to samples treated with placebo. Test–retest analysis of beta diversity indicated that bacterial compositions without pharmacological intervention exhibited only minor differences, although individual variations were noticeable as illustrated in Figure 3.

### Alpha diversity

**Figure 4:**
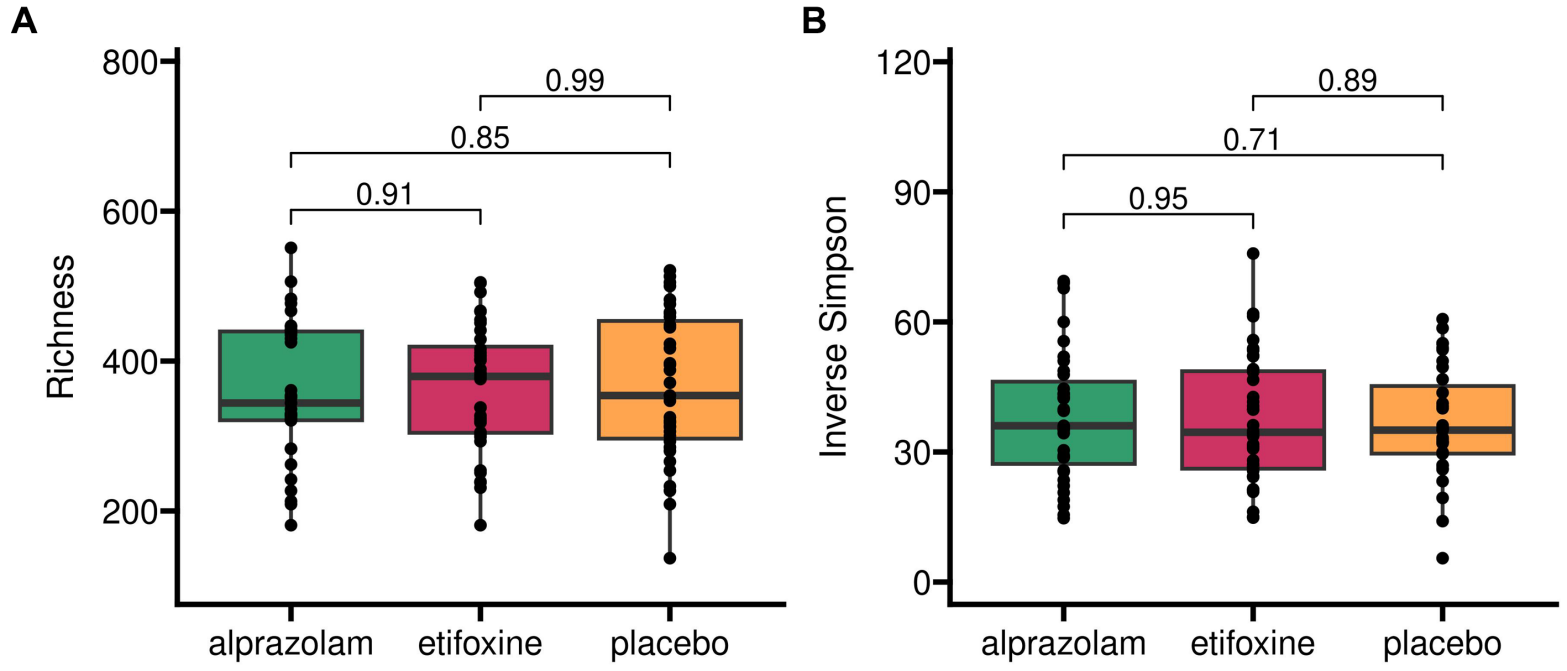
Alpha diversity of gut microbiome in treatment groups across complete trial. Two different indices for alpha diversity are shown to account for microbial richness alone (panel **A**) and proportional abundance (*Inverse Simpson*, panel **B**).

Microbiome alpha diversity indices (Observed Species, Inverse Simpson, Shannon, Hill) across the trial (Figure 4) did not vary between treatments, sequences and periods (i.e., time-points). Observed species (= Richness) across the full study design did not yield any significant differences between medications with *F*(2, 66.1) = 0.16 (*p* = 0.853), sequences with *F*(5, 30.0) = 1.12 (*p* = 0.371) and time-points with *F*(2, 66.1) = 0.51 (*p* = 0.606). Similarly, the Inverse Simpson index did not show any relevant effects for medications with *F*(2, 66.7) = 0.31 (*p* = 0.731), sequences with *F*(5, 30.2) = 1.49 (*p* = 0.222) and time-points with *F*(2, 66.7) = 0.60 (*p* = 0.553).

Alpha diversity grouped into sequence groups over time-points (periods) across the complete trial is shown in Supplementary Figure S1.

### Beta diversity

#### Intra-individual beta diversity over time

Firstly, beta diversity indices were calculated restricted to individual participants over treatment periods as a time series beginning with an initial baseline measurement. This individualized perspective on beta diversity (Wagner et al., 2018; Lahti and Shetty, 2020) provided three beta indices per participant corresponding to each of their treatment periods. This approach enabled the continued deployment of our standard model design for analysis.

**Figure 5:**
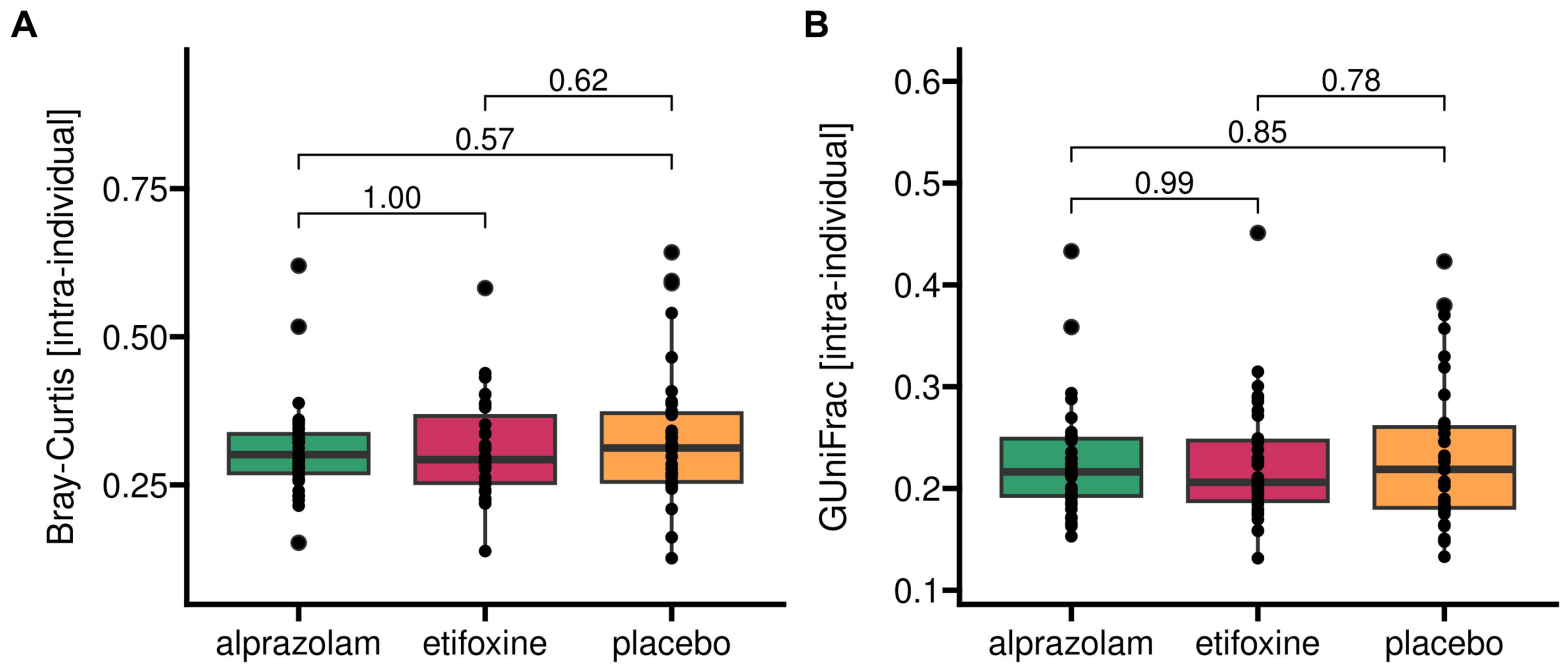
Beta diversity of gut microbiome as measured within each participant over treatment periods. Two different indices for beta diversity are shown. *Bray-Curtis* (panel **A**) accounts for dissimilarities on zOTU-levels and weighs these according to their relative abundances. *Generalized UniFrac* (panel **B**) additionally incorporates phylogenetic distances originating from genetic sequence dissimilarities. Generalized UniFrac with alpha = 1.0 corresponds to a conventional weighted UniFrac representation. Here, keeping the default of alpha = 0.5 reduces the weights of highly abundant species.

Microbiome beta diversity as measured within participants between time periods within their sequence group (Figure 5) did not vary between treatments, sequence groups and time-points. Specifically, Bray-Curtis_[intra-individual]_ did not show any significant effects for medication with *F*(2, 66) = 0.64 (*p* = 0.531), sequence groups with *F*(5, 29) = 0.33 (*p* = 0.890) and time-points with *F*(2, 66) = 0.79 (*p* = 0.458). Similarly, generalized UniFrac_[intra-individual]_ with alpha = 0.5 did not show any relevant effects for medication with *F*(2, 66) = 0.25 (*p* = 0.776), sequence groups with *F*(5, 29) = 0.30 (*p* = 0.908) and time-points with *F*(2, 66) = 1.21 (*p* = 0.304).

Intra-individual beta diversity grouped into sequence groups over time-points (periods) is shown in Supplementary Figure S2.

#### Beta diversity between all study samples

**Figure 6:**
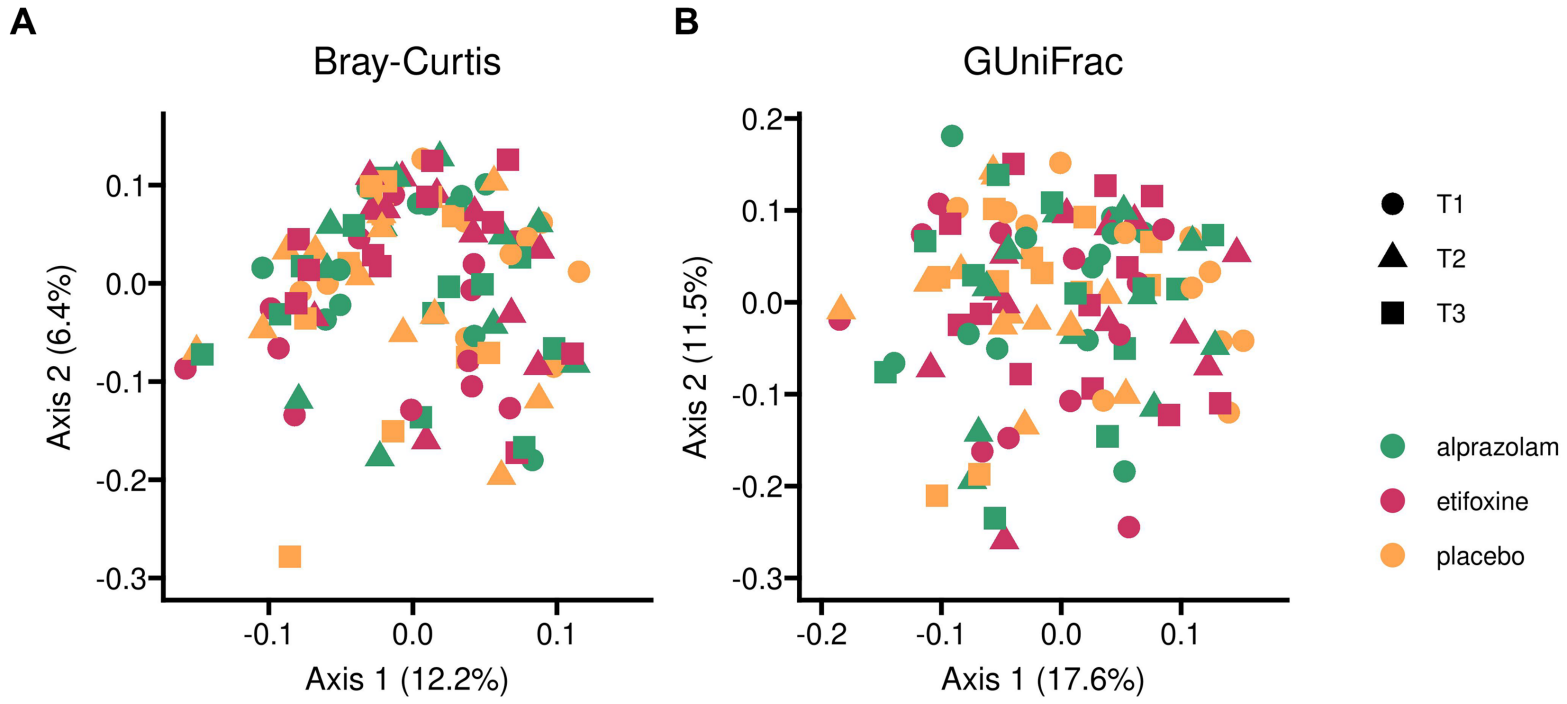
Beta diversity of gut microbiome among all samples across complete trial. Two different indices for beta diversity are shown. *Bray-Curtis* (panel **A**) accounts for dissimilarities on zOTU-levels and weighs these according to their relative abundances. *Generalized UniFrac* (panel **B**) additionally incorporates phylogenetic distances originating from genetic sequence dissimilarities. Generalized UniFrac with alpha = 1.0 corresponds to a conventional weighted UniFrac representation. Here, keeping the default of alpha = 0.5 reduces the weights of highly abundant species. Treatment groups and periods (time-points) within the balanced Williams design are differentiated by color and symbol shapes.

Secondly, analysis of beta diversity among all samples across the complete trial is shown in Figure 6. In this scenario, multivariate analyses are typically used as the standard in the analysis of microbial beta diversity. For this approach, two different algorithms were deployed for increased reliability. Both permutational multivariate analysis of variance (PERMANOVA) using distance matrices and distance-based redundancy analyses showed significant differences in beta diversity between randomized sequence groups (see individual results below), plus PERMANOVA multilevel pairwise comparisons confirmed the effect, while inferences with pairwise comparisons for all levels of a categorical variable by redundancy analysis revealed only borderline effects. Moreover, testing for homogeneity of group dispersions between randomized sequence groups showed borderline effects (*F*(5) = 2.13 (*p* = 0.068)), which did not reveal any significant group differences in post-hoc testing, and which seemed to be caused by borderline differences between sequences AEP and EAP (*p* = 0.06).

For adonis2, only fixed effects of the full linear mixed model were used. Bray-Curtis indices yielded no effects between treatments with *F*(2) = 0.45 (*p* = 1.000), but for sequences with *F*(5) = 2.65 (*p* = 0.001) and, again, none for time-points with *F*(2) = 0.29 (*p* = 1.000). Pairwise comparisons between sequences for Bray-Curtis distances confirmed significant differences for all group comparisons (*p* < 0.02). Generalized UniFrac indices yielded no effects between treatments with *F*(2) = 0.54 (*p* = 0.999), but, again, for sequences with *F*(5) = 2.36 (*p* = 0.001) and none for time-points with *F*(2) = 0.55 (*p* = 0.995). Pairwise comparisons between sequences for Generalized UniFrac distances showed 7 of 15 significant comparisons, mostly in group comparisons with sequences AEP, EAP and APE.

Db-RDA was used with our full linear mixed model. Bray-Curtis indices yielded no effects between treatments with *F*(2) = 0.67 (*p* = 1.000), but for sequences with *F*(5) = 2.08 (*p* = 0.001) and none for time-points with *F*(2) = 0.59 (*p* = 1.000). Generalized UniFrac indices yielded no effects between treatments with *F*(2) = 0.75 (*p* = 0.994), but for sequences with *F*(5) = 2.13 (*p* = 0.001) and none for time-points with *F*(2) = 0.76 (*p* = 0.995). Pairwise comparisons between sequences for Bray-Curtis distances did not reveal any significant differences between sequences, with APE-PAE (*p* = 0.066) and EAP-PAE (*p* = 0.073) at borderline.

**Figure 7:**
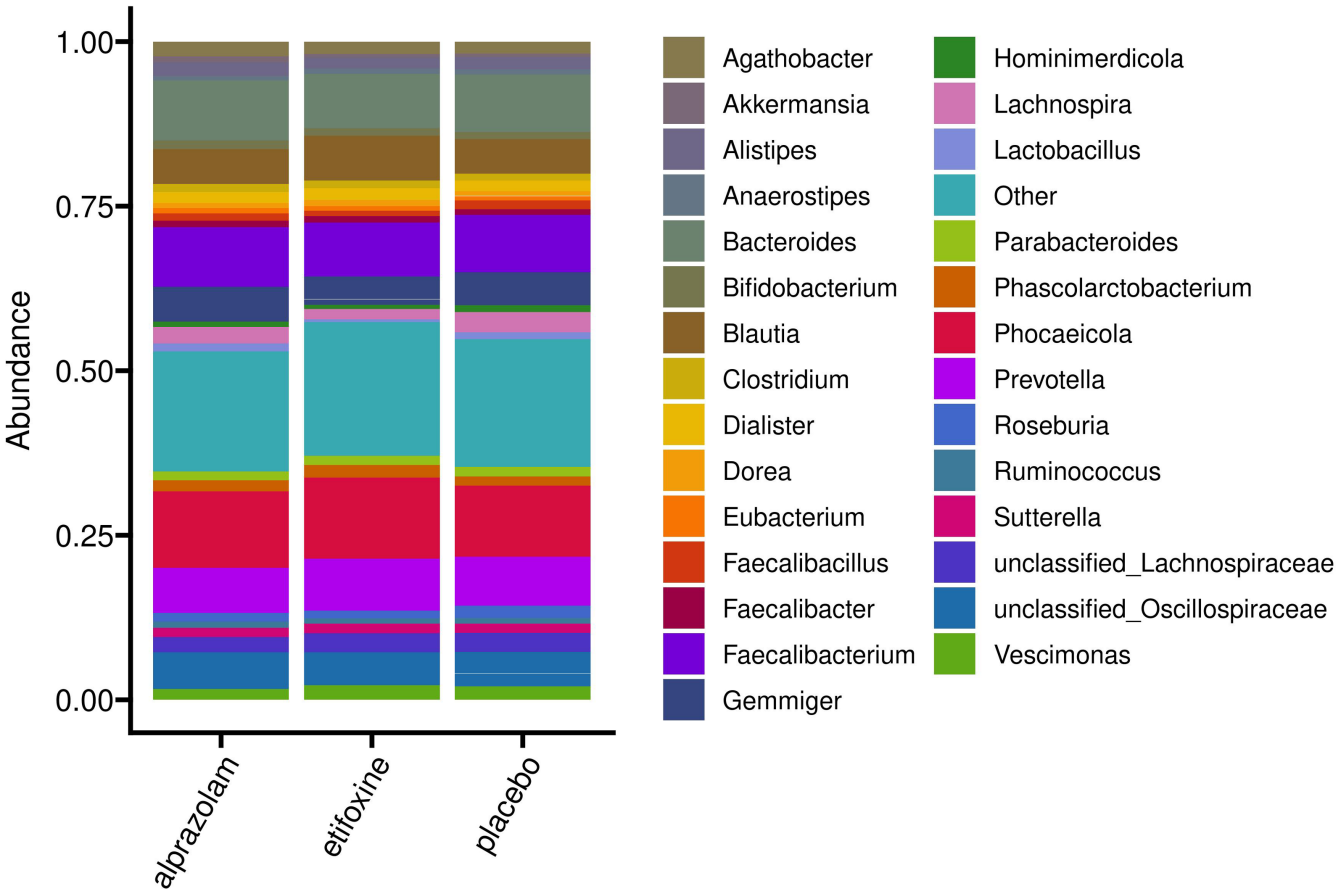
Mean relative abundances on genus level of the full study cohort (*N* = 36) grouped according to treatment. The 30 most abundant genera are shown explicitly, while remaining taxa are comprised in “Other.” Individual bacterial compositions for each participant are shown in Supplementary Figure S5.

Beta Diversity between all study samples grouped into sequence groups over time-points (periods) is shown in Supplementary Figure S3, and a grouping into participants over time in Supplementary Figure S4. Figure 7 shows an overview of mean relative abundances by presenting the most abundant genera averaged by treatment. Furthermore, Supplementary Table S1 provides an overview of the most abundant genera and their relative abundance at baseline for all participants before the trial started. Correspondingly, Supplementary Figure S5 shows relative abundance plots for each participant over time grouped along their treatment sequences.

### Differential abundance

**Figure 8:**
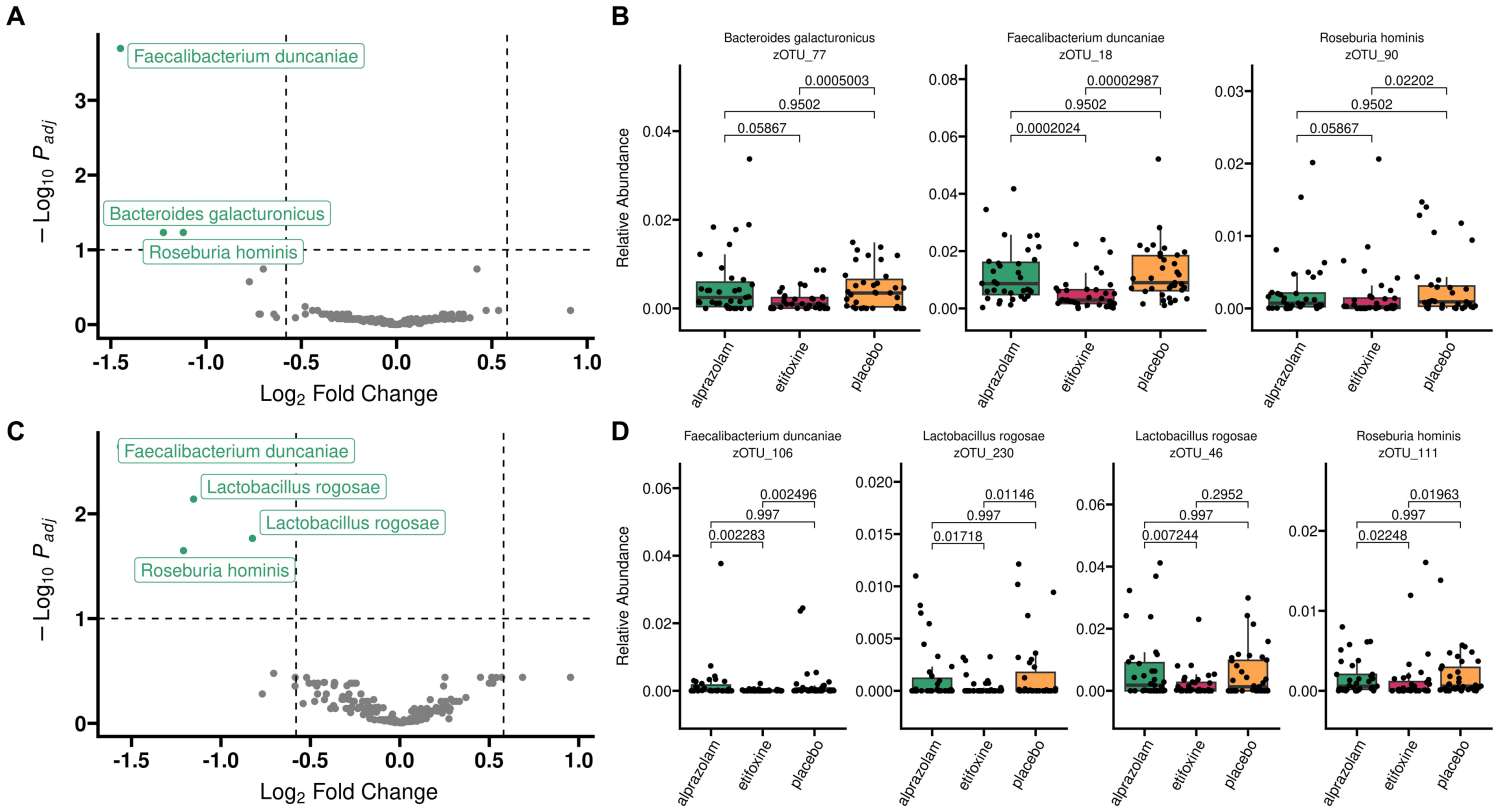
Differential abundance analysis with LinDA after applying a mean abundance filter of 0.1%. Alpha cutoff was relaxed to 10% to also show borderline zOTUs. Results are shown for two independent 16S rRNA gene regions V3V4 (*top*, panel **A,B**) and V1V3 (*bottom*, panel **C,D**). In both, the same three bacterial species show significant reductions of relative abundances after etifoxine treatment: *Faecalibacterium duncaniae*, *Bacteroides galacturonicus* (most likely *Lactobacillus rogosae*) and *Roseburia hominis.* Dashed lines were added in Volcano plots (panels **A,C**) to indicate an alpha cutoff of 10% (horizontal dashed line) and log2-fold-changes of 1.5 in both directions (vertical dashed lines). zOTUs below alpha of 10% are shown in gray color. Data for volcano plots originate from LinDA analysis which is optimized for handling zero-inflated absolute abundances.

Differential abundance analysis of V3V4 microbiome sequencing data (Figure 8, panels A,B) was performed with two methods [LinDA: *Linear Model for Differential Abundance Analysis of High-dimensional Compositional Data* (Zhou et al., 2022) and MaAsLin2: *Multivariable Association Discovery in Population-scale Meta-omics Studies* (Mallick et al., 2021)] and confirmed in independent sequencing results of the V1V3 hypervariable region (Figure 8, panels C,D).

Both methods consistently returned two zOTUs (18 and 90) in V3V4 with reduced abundances after administration of etifoxine (zOTU 18: log2FC = −1.45, zOTU 90: log2FC = −1.12), while LinDa returned an additional zOTU (77) as significant (log2FC = −1.22). Taxonomy assignment via LTP_06_2022 database with a mean abundance cutoff of 0.1% yielded *Faecalibacterium duncaniae* (zOTU 18, p_[LinDA]_ = 0.000202, p_[MaAsLin2]_ = 0.000198) and *Roseburia hominis* (zOTU 90, p_[LinDA]_ = 0.058673, p_[MaAsLin2]_ = 0.018641). LinDA additionally yielded *Bacteroides galacturonicus* (zOTU 77, p_[LinDA]_ = 0.058673, p_[MaAsLin2]_ = 0.218908). The latter (*Bacteroides galacturonicus*) may most likely be *Lactobacillus rogosae* according to confirmatory matching with SmartGene IDNS 16S rDNA Eubacteria database in combination with “Problematic Species”: *Bacteroides galacturonicus* accompanying LTP_12_2020 database description (Ludwig et al., 2021).

## Discussion

This study presents the first human gut microbiome data with etifoxine intervention. In a balanced Williams crossover design, 36 healthy males received etifoxine, alprazolam and placebo for 5 days each. While microbial alpha and beta diversity did not show any apparent changes between treatments, differential abundance analyses revealed three bacterial species that were significantly reduced after short-term etifoxine administration, *Faecalibacterium duncaniae*, *Roseburia hominis* and *Lactobacillus rogosae* (i.e., *Bacteroides galacturonicus*). Among these three, effects were largest and most stable for *Faecalibacterium duncaniae*.

It is not uncommon for gut microbiome alpha diversities to show no relevant differences in clinical neuroscience research (Plassais et al., 2021; Kovtun et al., 2022). Moreover, even differences previously reported in beta diversity may not be as significant as originally claimed (Weiss et al., 2017; Schloss, 2018). Furthermore, there is growing evidence that a persistent lack of standardization in pre-analytical procedures (Sinha et al., 2017) and analysis pipelines over the last 15 years have substantially contributed to an increasing number of irreproducible results (Nearing et al., 2022). There may be several reasons for the lack of differences in alpha and beta diversity in our study.

A sample size of 36 participants for a microbiome investigation may appear rather small. However, several other microbiome studies have gathered microbiome samples, for example from depressed patients, with a similar range of sample sizes (Barandouzi et al., 2020). This putative deficiency in statistical power may be related to the enormous challenge of power estimation for microbiome studies (Debelius et al., 2016). The reasons for this are numerous and reach from the workbench to the bioinformatical pipeline. Hence, more recent statistical approaches additionally try to increase the power of microbiome analyses with already existing data after trials have ended (Jouffret et al., 2021; Martino et al., 2022).

Furthermore, our test–retest measurements for alpha diversity (Figure 2) and beta diversity (Figure 3) show a certain variance in within-subjects data without experimental intervention. For reasons of feasibility and etiquette, participants were unable to deliver samples from a specific time of day (Nobs et al., 2019), and their complete stool output was not homogenized before sampling (Zmora et al., 2018; Jones et al., 2021). Furthermore, there were about 5 days between baseline and first treatment (with placebo) measurements during which participants had experienced various daily life influences (Falony et al., 2016; Uhr et al., 2019) which could not be controlled for in a common research setting like ours. Therefore, we consider this magnitude of variance in test–retest data as rather typical.

Our study design, a uniform and balanced Williams crossover design (Williams, 1949) with three treatments over 5 days each, was carefully selected for this investigation. However, it may have limitations in terms of producing contrasts between treatments. On the one hand, we opted to include placebo and therefore did not choose stricter crossover designs such as a strongly balanced or even a strongly balanced and uniform crossover design with just two treatments in two or four sequences, respectively. On the other hand, there is only limited public data available regarding the pharmacokinetics of the two compounds used in this study, particularly etifoxine. Therefore, we selected a conservative definition for wash-out periods based on the limited pharmacokinetic data available. However, as Owen et al. mentioned recently (Owen et al., 2022), this approach does not account for the potential of more complex and long-term effects of drugs like etifoxine.

Moreover, the treatment period of only 5 days was short. There were several reasons for this choice of treatment duration. First, this duration is representative of a naturalistic treatment setting. Second, other researchers have reported significant clinical effects of etifoxine within 7 days (Stein, 2015). And third, in contrast to etifoxine, alprazolam – like all other benzodiapezepines –unfortunately foster development of tolerance and physical dependence, quickly. Therefore, 5 days of administration in healthy subjects was an ethical choice. The exposure time only allowed us to investigate short-term effects of etifoxine. Effects with a greater time lag further downstream would need to be captured during longer interventional periods in future human studies. Ibrahim et al. (2020) administered etifoxine to mice for 15 days at an intraperitoneal dose of 50 mg/kg. Dose translations between animals and humans remain a disputed topic. Recommendations based on body surface area normalization as published by Food and Drug Administration (FDA) (2005) and Reagan-Shaw et al. (2008) suggest conversion factors for humans to mice and rats of 12.3 and 6.2, respectively. Using a conversion factor of 12.3 with 50 mg/kg in mice would correspond to a human etifoxine dose of 285 to 325 mg/day depending on the reference weight (70 or 80 kg, respectively). This is similar to the initial human experimental doses of 300 mg/day (Córsico et al., 1976). However, the recommended daily etifoxine dose today is 150–200 mg (Servant et al., 1998; Choi and Kim, 2015).

The traditional method of gut microbiome sampling relies on collecting rectal stool output. However, this fails to capture the complex dynamics throughout the entire gastrointestinal microbiome (Zmora et al., 2018). In a biodistribution study with alprazolam (Banks et al., 1992), the intestines of small animals were examined as a whole including their contents. The authors noted an increased intestinal uptake, especially an increase over time, and they postulated that biliary excretion and entero-hepatic recirculation may play a role in this context. Beyond that, other important considerations are where in the gut these drugs are primarily taken up, and how their metabolites are distributed along the gut, including their residence times and in which sections of the gut their effects on the microbiome may be greatest. Therefore, traditional rectal stool sampling can only reveal a small part of an interesting story along the gut.

In all of our analyses, we observed only a few significant differences or borderline effects between randomized sequences, such as in microbial beta diversity. Since our sequences consisted of only six participants, we believe that these effects are likely due to between-subjects effects (Supplementary Figure S4) indicating post-randomization confounding between sequences (Miettinen and Cook, 1981; Rochon, 1996). However, our model design took randomization into account. Furthermore, a balanced Williams design was used to minimize confounding and to improve the evaluation of differences between treatments. In general, the primary objective of this design is to compare the effects of individual treatments, not the sequences themselves (Kenward and Jones, 2007). Correspondingly, we ensured that microbiome parameters were restricted to a within-subject level, for example, by defining an appropriate intra-individual beta diversity measure. Therefore, we assume that this type of confounding did not affect the results of our study.

Etifoxine is a TSPO ligand with relevant uptake in the intestinal tract, providing motivation for looking at the bacterial perspective on TSPO. Interest in TSPO initially arose from its discovery as a high-affinity binding receptor in diazepam binding assays with homogenized rat brain (Braestrup and Squires, 1977). It was subsequently referred to as a peripheral-type benzodiazepine receptor (PBR). It is now evident that the large family of tryptophan-rich sensory proteins (TSPOs) is well-conserved in evolution, and members of this family have been identified in various species across all kingdoms, including animals, plants, fungi, bacteria and archaea (Hiser et al., 2021). Thus, TSPO is regarded as a multifunctional housekeeping gene (Gavish and Veenman, 2018). Bacterial TspO may be involved in various metabolic processes such as response to oxidative stress, regulation of cell cycle and growth, porphyrin transport, heme metabolism or cell adhesion (Veenman et al., 2016). Moreover, it has also been described as a nonessential gene (Batoko et al., 2015), which is involved in regulating photosynthetic gene expression in response to oxygen and light conditions, primarily upregulated during oxidative stress which caused bacteria to switch from aerobic to anaerobic metabolism (Yeliseev et al., 1997). In this context, it supports the endosymbiontic hypothesis for mitochondria. The binding of endogenous ligands such as tetrapyrroles including protoporphyrins, as well as the binding of well-known synthetic ligands such as PK11195 to bacterial TSPO has been demonstrated (Leneveu-Jenvrin et al., 2014; Hiser et al., 2021). However, to date, no data are available on the quality of binding or effects of etifoxine on bacterial TSPO, nor on the prevalence and distribution of TSPO expression among individual intestinal bacteria. Nevertheless, a direct interaction seems likely and, thus, effects on regulatory pathways or growth rates of certain gut bacteria cannot be excluded.

The role of etifoxine as a GABA-A receptor ligand in the gut is unclear. Yet, the enteric nervous system is rich in a variety of GABA-A receptors (Seifi et al., 2014) making a direct interaction of etifoxine with the enteric nervous system likely. Some of these receptors are directly involved in gastrointestinal motility (Hosie et al., 2019). However, the diversity and different spread of GABA-A receptors provides for high complexity in which GABA-induced effects depend on animal species, region of the gastrointestinal tract and the GABA receptors involved (Auteri et al., 2015). In contrast to the function of GABA-A receptors in the central nervous system, activation of enteric GABA-A receptors causes excitatory effects and in humans probably increases contractile motor activity (Auteri et al., 2015). This might cause opposing effects of etifoxine depending on its binding to central or peripheral GABA-A receptors. Furthermore, GABA-signaling is present in bacterial communities (Guthrie and Nicholson-Guthrie, 1989) and hence, etifoxine may possibly exert direct effects via bacterial GABA receptors as well (Quillin et al., 2021). For the moment, this complexity and lacking data hinder reasonable hypotheses on etifoxine-mediated GABA-A transmission in the gut.Differential abundance analyses between treatments identified several zOTUs that were significantly reduced following etifoxine administration. These features were assigned to the bacterial species *Faecalibacterium duncaniae*, *Lactobacillus rogosae/Bacteroides galacturonicus* and *Roseburia hominis*. These species are known as common commensals of the human gut. Interestingly, *Faecalibacterium* and *Roseburia* species are among the most abundant butyrate producing bacteria in the human gut (Barcenilla et al., 2000; Hold et al., 2003).

Butyrate is solely produced by microbes in the human body. It is a four-carbon short-chain fatty acid (SCFA), which is mainly produced by bacterial fermentation of undigested carbohydrates or lysine in the human colon. It is a major source of energy for colonocytes in the gut (Tan et al., 2014) and exerts multiple systemic effects via different mechanisms, such as inhibiting histone deacetylase activity, thereby altering host gene expression or signaling through G-protein-coupled receptors (Davie, 2003). Its anti-inflammatory properties, for example, inhibiting proinflammatory cytokines (Aguilar et al., 2014) or promoting differentiation of regulatory T-cells (Singh et al., 2014), are among the many important characteristics of butyrate. Thus, it is considered a beneficial molecule in maintaining intestinal health.

Direct effects of butyrate on the brain are still unclear. On the one hand, it may stabilize blood–brain barrier function, as demonstrated in germ-free mice displaying increased permeability of the blood–brain barrier (Braniste et al., 2014), which was then mended by butyrate. On the other hand, physiological butyrate levels in the brain are likely to be very low (Kim et al., 2013), and experimental dosages of butyrate, which are far beyond physiological levels, may constitute a pharmacological stressor (Gagliano et al., 2014).

In our study, *Faecalibacterium duncaniae* showed a stable effect across different methods (LinDA, Maaslin2) and different gene regions of the 16S rRNA gene (V3V4 and V1V3). Very recently, it was proposed as a novel species and was split off from *Faecalibacterium prausnitzii* by taxonomic reclassification based on whole genome and phenotypic comparisons (Sakamoto et al., 2022). No published studies to date have examined the role of *Faecalibacterium duncaniae* in the context of health and disease. Past studies describing *Faecalibacterium prausnitzii* and its potential role in the intestinal microbiome are likely based on several, now distinguishable, species, including *Faecalibacterium duncaniae*. Both species are genetically very similar and they do not differ in the spectrum and concentrations of major fermentation products in growing cultures. Butyrate is their major fermentation product, while formate and lactate are excreted only in low amounts (Sakamoto et al., 2022). For example, administration of *Faecalibacterium prausnitzii* to rats had preventive and therapeutic effects on chronic unpredictable mild stress-induced depression-like and anxiety-like behavior (Hao et al., 2019).

*Roseburia hominis* is a strictly anaerobic bacterium that frequently inhabits the human gut and utilizes acetate and dietary mono- or disaccharides to produce mainly butyrate and formate (Duncan et al., 2006). When germ-free mice were mono-colonized with *Roseburia hominis*, immunomodulatory capacities were apparent, for example by expansion of regulatory T-cells and by enhancing tight junction integrity, thus strengthening gut barrier function (Patterson et al., 2017). In neurobiological investigations, germ-free rats were mono-colonized with *Roseburia hominis*, which reduced microglial activation and proinflammatory cytokines (Song et al., 2022). Furthermore, in patients with Alzheimer’s disease, lower abundances of *Roseburia hominis* were associated with both higher amyloid and lower phosphorylated-tau levels (Verhaar et al., 2021), which supports a potential role along the gut–brain axis.

Besides the two species described above, *Bacteroides galacturonicus* (assignment in V3V4 region) and *Lactobacillus rogosae* (assignment in V1V3 region) were among the significantly reduced features in participants after etifoxine treatment. Both species are nearly identical based on their 16S rRNA gene sequences and cannot be discriminated from each other in the V3V4 region. Moreover, it is currently being debated whether both species are valid in the *List of Prokaryotic names with Standing in Nomenclature (LPSN)* (Parte et al., 2020) or whether these species may be reclassified altogether into a new genus of *Lachnospiraceae* (Tindall, 2014; Ludwig et al., 2021). Since the actual status of these species is unclear and reclassification of both type strains of *Lactobacillus rogosae* as well as *Bacteroides galacturonicus* is being considered, it is not feasible to assess the impact of these species until further clarification. Initial reports on these species described them both as being capable of degrading pectin and related compounds, mainly towards acetate and formate (Jensen and Canale-Parola, 1986; Felis et al., 2004).

All three species described above are considered beneficial members of a healthy gut microbiome. One of their strong metabolic links could be acetate (Duncan et al., 2002), which is excreted by pectinophilic species like *Lactobacillus rogosae* and fed to butyrate producers like *Faecalibacterium duncaniae* and *Roseburia hominis*. It is important to note that the gut consists of very complex microbial networks, located in equally complex metabolic interdependencies between a multitude of subcommunities (Chen et al., 2020). The effect of an external substance like etifoxine on a single bacterium may therefore result in changes of many interdependent species.

The observed differences in abundance in this study are subtle and the physiological context of these small reductions after etifoxine treatment remains unclear. Furthermore, given the complex and intricate interactions between the microbiota and the host, it is uncertain whether the observed reductions are linked to direct or indirect mechanisms of etifoxine, and therefore, definitve conclusions about etifoxine effects on these species cannot be made. Nevertheless, recent evidence on the impact of sex-specific gut steroids on the gut microbiome in rats supports our findings in humans. Diviccaro et al. demonstrated that *Roseburia* were inversely associated with allopregnanolone, pregnenolone, isoallopregnanolone, progesterone, dihydroprogesterone and testosterone (Diviccaro et al., 2022). Additionally, it appears likely that etifoxine elevated these gut steroids, presumably through the enteric nervous system (Giatti et al., 2020).

In conclusion, our study shows that short-team treatment with etifoxine may induce subtle alterations in human gut microbiome composition of healthy male participants. These might be related to the unique pharmacological profile of etifoxine and underline the importance of the gut-brain axis for health and disease.

## Data availability statement

The datasets for this article are not publicly available due to concerns regarding participant anonymity. Requests to access the datasets should be directed to the corresponding author.

## Ethics statement

The studies involving humans were approved by Ethikkommission bei der Universität Regensburg, 93040 Regensburg, Germany. The studies were conducted in accordance with the local legislation and institutional requirements. The participants provided their written informed consent to participate in this study.

## Author contributions

AM and AH conceptualized and designed the microbiome investigation of this study, curated the databases, reviewed experimental design considerations, performed all analyses, and wrote the manuscript. TB contributed to design of the microbiome investigation and reviewed analysis design. MR recruited and screened subjects. CN, JS, and RR contributed to conception and design of the overall study and to funding acquisition. AG supported the complete microbiome analysis pipeline. All authors contributed to the article and approved the submitted version.

## References

[ref1] Aguilar E. C. Leonel A. J. Teixeira L. G. Silva A. R. Silva J. F. Pelaez J. M. N.. (2014). Butyrate impairs atherogenesis by reducing plaque inflammation and vulnerability and decreasing NFκB activation. Nutr Metab Cardiovasc Dis NMCD. 24, 606–613. doi: 10.1016/j.numecd.2014.01.002, PMID: 24602606

[ref2] Aitken R. C. (1969). Measurement of feelings using visual analogue scales. Proc. R. Soc. Med. 62, 989–993. doi: 10.1177/003591576906201005 4899510 PMC1810824

[ref3] Arumugam M. Raes J. Pelletier E. Le Paslier D. Yamada T. Mende D. R.. (2011). Enterotypes of the human gut microbiome. Nature 473, 174–180. doi: 10.1038/nature09944, PMID: 21508958 PMC3728647

[ref4] Auteri M. Zizzo M. G. Serio R. (2015). GABA and GABA receptors in the gastrointestinal tract: from motility to inflammation. Pharmacol. Res. 93, 11–21. doi: 10.1016/j.phrs.2014.12.001 25526825

[ref5] Baayen R. H. Davidson D. J. Bates D. M. (2008). Mixed-effects modeling with crossed random effects for subjects and items. J. Mem. Lang. 59, 390–412. doi: 10.1016/j.jml.2007.12.005

[ref6] Banati R. B. Middleton R. J. Chan R. Hatty C. R. Kam W. W. Y. Quin C.. (2014). Positron emission tomography and functional characterization of a complete PBR/TSPO knockout. Nat. Commun. 5:5452. doi: 10.1038/ncomms6452 25406832 PMC4263137

[ref7] Banks W. R. Yamakita H. Digenis G. A. (1992). Metabolism and distribution of 1-[14C]alprazolam in rats. J. Pharm. Sci. 81, 797–801. doi: 10.1002/jps.2600810815, PMID: 1403726

[ref8] Barandouzi Z. A. Starkweather A. R. Henderson W. A. Gyamfi A. Cong X. S. (2020). Altered composition of gut microbiota in depression: a systematic review. Front. Psych. 11:541. doi: 10.3389/fpsyt.2020.00541, PMID: 32587537 PMC7299157

[ref9] Barcenilla A. Pryde S. E. Martin J. C. Duncan S. H. Stewart C. S. Henderson C.. (2000). Phylogenetic relationships of butyrate-producing bacteria from the human gut. Appl. Environ. Microbiol. 66, 1654–1661. doi: 10.1128/AEM.66.4.1654-1661.2000, PMID: 10742256 PMC92037

[ref10] Bastiaanssen T. F. S. Cussotto S. Claesson M. J. Clarke G. Dinan T. G. Cryan J. F. (2020). Gutted! Unraveling the role of the microbiome in major depressive disorder. Harv. Rev. Psychiatry 28, 26–39. doi: 10.1097/HRP.0000000000000243, PMID: 31913980 PMC7012351

[ref11] Batoko H. Veljanovski V. Jurkiewicz P. (2015). Enigmatic translocator protein (TSPO) and cellular stress regulation. Trends Biochem. Sci. 40, 497–503. doi: 10.1016/j.tibs.2015.07.001, PMID: 26228316

[ref12] Benavides J. Quarteronet D. Imbault F. Malgouris C. Uzan A. Renault C.. (1983). Labelling of “peripheral-type” benzodiazepine binding sites in the rat brain by using [3H]PK 11195, an isoquinoline carboxamide derivative: kinetic studies and autoradiographic localization. J. Neurochem. 41, 1744–1750. doi: 10.1111/j.1471-4159.1983.tb00888.x, PMID: 6315880

[ref13] Boissier J. R. Simon P. Zaczinska M. Fichelle J. (1972). Etude psychopharmacologie expérimentale d’une nouvelle substante psychotrope, la 2-éthylamino-6-chloro-4-méthyl-4-phényl-4 H-3,1-benzoxazine. Therapie 27, 325–338. 5084676

[ref14] Braestrup C. Squires R. F. (1977). Specific benzodiazepine receptors in rat brain characterized by high-affinity (3H)diazepam binding. Proc. Natl. Acad. Sci. U. S. A. 74, 3805–3809. doi: 10.1073/pnas.74.9.3805, PMID: 20632 PMC431738

[ref15] Braniste V. Al-Asmakh M. Kowal C. Anuar F. Abbaspour A. Tóth M.. (2014). The gut microbiota influences blood-brain barrier permeability in mice. Sci. Transl. Med. 6:263ra158. doi: 10.1126/scitranslmed.3009759, PMID: 25411471 PMC4396848

[ref16] Chen L. Collij V. Jaeger M. van den Munckhof I. C. L. Vich Vila A. Kurilshikov A.. (2020). Gut microbial co-abundance networks show specificity in inflammatory bowel disease and obesity. Nat. Commun. 11:4018. doi: 10.1038/s41467-020-17840-y, PMID: 32782301 PMC7419557

[ref17] Choi Y. M. Kim K. H. (2015). Etifoxine for pain patients with anxiety. Korean J Pain. 28, 4–10. doi: 10.3344/kjp.2015.28.1.4, PMID: 25589941 PMC4293506

[ref18] Clayton A. H. Lasser R. Nandy I. Sankoh A. J. Jonas J. Kanes S. J. (2023). Zuranolone in major depressive disorder: results from MOUNTAIN-A phase 3, multicenter, double-blind, randomized, placebo-controlled trial. J. Clin. Psychiatry 84:22m14445. doi: 10.4088/JCP.22m14445 36811520

[ref19] Corpéchot C. Robel P. Axelson M. Sjövall J. Baulieu E. E. (1981). Characterization and measurement of dehydroepiandrosterone sulfate in rat brain. Proc. Natl. Acad. Sci. U. S. A. 78, 4704–4707. doi: 10.1073/pnas.78.8.4704, PMID: 6458035 PMC320231

[ref20] Córsico R. Moizeszowicz J. Bursuck L. Rovaro E. (1976). Evaluation of the psychotropic effect of etifoxine through pursuit rotor performance and GSR. Psychopharmacologia 45, 301–303. doi: 10.1007/BF00421144 1250945

[ref21] Costa B. Cavallini C. Da Pozzo E. Taliani S. Da Settimo F. Martini C. (2017). The anxiolytic Etifoxine binds to TSPO Ro5-4864 binding site with long residence time showing a high Neurosteroidogenic activity. ACS Chem. Neurosci. 8, 1448–1454. doi: 10.1021/acschemneuro.7b00027, PMID: 28362078

[ref22] Cottin J. Gouraud A. Jean-Pastor M. J. Dautriche A. D. Boulay C. Geniaux H.. (2016). Safety profile of etifoxine: a French pharmacovigilance survey. Fundam. Clin. Pharmacol. 30, 147–152. doi: 10.1111/fcp.12169, PMID: 26588183

[ref23] Davie J. R. (2003). Inhibition of histone deacetylase activity by butyrate. J. Nutr. 133, 2485S–2493S. doi: 10.1093/jn/133.7.2485S 12840228

[ref24] Debelius J. Song S. J. Vazquez-Baeza Y. Xu Z. Z. Gonzalez A. Knight R. (2016). Tiny microbes, enormous impacts: what matters in gut microbiome studies? Genome Biol. 17:217. doi: 10.1186/s13059-016-1086-x, PMID: 27760558 PMC5072314

[ref25] Diaz Heijtz R. Wang S. Anuar F. Qian Y. Bjorkholm B. Samuelsson A.. (2011). Normal gut microbiota modulates brain development and behavior. Proc Natl Acad Sci U S A 108, 3047–3052. doi: 10.1073/pnas.1010529108, PMID: 21282636 PMC3041077

[ref26] Dinan T. G. Cryan J. F. (2012). Regulation of the stress response by the gut microbiota: implications for psychoneuroendocrinology. Psychoneuroendocrinology 37, 1369–1378. doi: 10.1016/j.psyneuen.2012.03.007, PMID: 22483040

[ref27] Diviccaro S. Caputi V. Cioffi L. Giatti S. Lyte J. M. Caruso D.. (2021). Exploring the impact of the microbiome on neuroactive steroid levels in germ-free animals. Int. J. Mol. Sci. 22:12551. doi: 10.3390/ijms222212551, PMID: 34830433 PMC8622241

[ref28] Diviccaro S. Falvo E. Piazza R. Cioffi L. Herian M. Brivio P.. (2023). Gut microbiota composition is altered in a preclinical model of type 1 diabetes mellitus: influence on gut steroids, permeability, and cognitive abilities. Neuropharmacology 226:109405. doi: 10.1016/j.neuropharm.2022.109405, PMID: 36572179

[ref29] Diviccaro S. Giatti S. Cioffi L. Falvo E. Herian M. Caruso D.. (2022). Gut inflammation induced by finasteride withdrawal: therapeutic effect of Allopregnanolone in adult male rats. Biomol. Ther. 12:1567. doi: 10.3390/biom12111567, PMID: 36358917 PMC9687671

[ref30] do Rego J. L. Vaudry D. Vaudry H. (2015). The non-benzodiazepine anxiolytic drug etifoxine causes a rapid, receptor-independent stimulation of neurosteroid biosynthesis. PLoS One 10:e0120473. doi: 10.1371/journal.pone.0120473, PMID: 25785994 PMC4364751

[ref31] Duncan S. H. Aminov R. I. Scott K. P. Louis P. Stanton T. B. Flint H. J. (2006). Proposal of *Roseburia faecis* sp. nov., *Roseburia hominis* sp. nov. and *Roseburia inulinivorans* sp. nov., based on isolates from human faeces. Int. J. Syst. Evol. Microbiol. 56, 2437–2441. doi: 10.1099/ijs.0.64098-0, PMID: 17012576

[ref32] Duncan S. H. Barcenilla A. Stewart C. S. Pryde S. E. Flint H. J. (2002). Acetate utilization and butyryl coenzyme a (CoA):acetate-CoA transferase in butyrate-producing bacteria from the human large intestine. Appl. Environ. Microbiol. 68, 5186–5190. doi: 10.1128/AEM.68.10.5186-5190.2002, PMID: 12324374 PMC126392

[ref33] European Medical Agency. EMA Concludes Review of Anxiety Medicine Stresam (Etifoxine). European Medicines Agency; (2022). Available at: https://www.ema.europa.eu/en/documents/referral/etifoxine-containing-medicinal-products-article-31-referral-ema-concludes-review-anxiety-medicine_en-0.pdf (Accessed February 22, 2023).

[ref34] Falony G. Joossens M. Vieira-Silva S. Wang J. Darzi Y. Faust K.. (2016). Population-level analysis of gut microbiome variation. Science 352, 560–564. doi: 10.1126/science.aad3503, PMID: 27126039

[ref35] Felis G. E. Torriani S. Dellaglio F. (2004). The status of the species *Lactobacillus rogosae* Holdeman and Moore 1974. Request for an opinion. Int. J. Syst. Evol. Microbiol. 54, 1903–1904. doi: 10.1099/ijs.0.63099-0 15388761

[ref36] Food and Drug Administration (FDA). (2001). Statistical Approaches to Establishing Bioequivalence. U.S. Department of Health and Human Services, Food and Drug Administration, Center for Drug Evaluation and Research (CDER). Available at: https://www.fda.gov/media/70958/download (Accessed February 22, 2023).

[ref37] Food and Drug Administration (FDA). (2005). Estimating the Maximum Safe Starting Dose in Initial Clinical Trials for Therapeutics in Adult Healthy Volunteers. U. S. Department of Health and Human Services, Food and Drug Administration, Center for Drug Evaluation and Research (CDER). Available at: https://www.fda.gov/media/72309/download (Accessed February 26, 2023).

[ref38] Fujii J. Inotsume N. Nakano M. (1987). Degradation of bromazepam by the intestinal microflora. Chem Pharm Bull (Tokyo). 35, 4338–4341. doi: 10.1248/cpb.35.4338, PMID: 2893665

[ref39] Gagliano H. Delgado-Morales R. Sanz-Garcia A. Armario A. (2014). High doses of the histone deacetylase inhibitor sodium butyrate trigger a stress-like response. Neuropharmacology 79, 75–82. doi: 10.1016/j.neuropharm.2013.10.031, PMID: 24212060

[ref40] Gavish M. Veenman L. (2018). Regulation of mitochondrial, cellular, and organismal functions by TSPO. Adv Pharmacol San Diego Calif. 82, 103–136. doi: 10.1016/bs.apha.2017.09.004 29413517

[ref41] Giatti S. Diviccaro S. Serafini M. M. Caruso D. Garcia-Segura L. M. Viviani B.. (2020). Sex differences in steroid levels and steroidogenesis in the nervous system: Physiopathological role. Front. Neuroendocrinol. 56:100804. doi: 10.1016/j.yfrne.2019.100804, PMID: 31689419

[ref42] Gunay A. Pinna G. (2022). The novel rapid-acting neurosteroid-based antidepressant generation. Curr Opin Endocr Metab Res. 24:100340. doi: 10.1016/j.coemr.2022.100340

[ref43] Gunduz-Bruce H. Silber C. Kaul I. Rothschild A. J. Riesenberg R. Sankoh A. J.. (2019). Trial of SAGE-217 in patients with major depressive disorder. N. Engl. J. Med. 381, 903–911. doi: 10.1056/NEJMoa1815981, PMID: 31483961

[ref44] Guthrie G. D. Nicholson-Guthrie C. S. (1989). Gamma-aminobutyric acid uptake by a bacterial system with neurotransmitter binding characteristics. Proc. Natl. Acad. Sci. U. S. A. 86, 7378–7381. doi: 10.1073/pnas.86.19.7378, PMID: 2552441 PMC298065

[ref45] Han W. Wang N. Han M. Ban M. Sun T. Xu J. (2022). Reviewing the role of gut microbiota in the pathogenesis of depression and exploring new therapeutic options. Front. Neurosci. 16:1029495. doi: 10.3389/fnins.2022.1029495, PMID: 36570854 PMC9772619

[ref46] Hao Z. Wang W. Guo R. Liu H. (2019). *Faecalibacterium prausnitzii* (ATCC 27766) has preventive and therapeutic effects on chronic unpredictable mild stress-induced depression-like and anxiety-like behavior in rats. Psychoneuroendocrinology 104, 132–142. doi: 10.1016/j.psyneuen.2019.02.025, PMID: 30844607

[ref47] Hiser C. Montgomery B. L. Ferguson-Miller S. (2021). TSPO protein binding partners in bacteria, animals, and plants. J. Bioenerg. Biomembr. 53, 463–487. doi: 10.1007/s10863-021-09905-4, PMID: 34191248 PMC8243069

[ref48] Hold G. L. Schwiertz A. Aminov R. I. Blaut M. Flint H. J. (2003). Oligonucleotide probes that detect quantitatively significant groups of butyrate-producing bacteria in human feces. Appl. Environ. Microbiol. 69, 4320–4324. doi: 10.1128/AEM.69.7.4320-4324.2003, PMID: 12839823 PMC165216

[ref49] Hosie S. Ellis M. Swaminathan M. Ramalhosa F. Seger G. O. Balasuriya G. K.. (2019). Gastrointestinal dysfunction in patients and mice expressing the autism-associated R451C mutation in neuroligin-3. Autism Res Off J Int Soc Autism Res. 12, 1043–1056. doi: 10.1002/aur.2127, PMID: 31119867 PMC6606367

[ref50] Hosie A. M. Wilkins M. E. da Silva H. M. A. Smart T. G. (2006). Endogenous neurosteroids regulate GABAA receptors through two discrete transmembrane sites. Nature 444, 486–489. doi: 10.1038/nature05324, PMID: 17108970

[ref51] Ibrahim K. S. Craft J. A. Biswas L. Spencer J. Shu X. (2020). Etifoxine reverses weight gain and alters the colonic bacterial community in a mouse model of obesity. Biochem. Pharmacol. 180:114151. doi: 10.1016/j.bcp.2020.114151 32679124

[ref52] Jensen N. S. Canale-Parola E. (1986). *Bacteroides pectinophilus* sp. nov. and *Bacteroides galacturonicus* sp. nov.: two pectinolytic bacteria from the human intestinal tract. Appl. Environ. Microbiol. 52, 880–887. doi: 10.1128/aem.52.4.880-887.1986, PMID: 3777933 PMC239131

[ref53] Jones B. Kenward M. G. (2014). Design and Analysis of Cross-Over Trials. 3rd Edn. Taylor & Francis Group: CRC Press.

[ref54] Jones J. Reinke S. N. Ali A. Palmer D. J. Christophersen C. T. (2021). Fecal sample collection methods and time of day impact microbiome composition and short chain fatty acid concentrations. Sci. Rep. 11:13964. doi: 10.1038/s41598-021-93031-z, PMID: 34234185 PMC8263620

[ref55] Jouffret V. Miotello G. Culotta K. Ayrault S. Pible O. Armengaud J. (2021). Increasing the power of interpretation for soil metaproteomics data. Microbiome. 9:195. doi: 10.1186/s40168-021-01139-1, PMID: 34587999 PMC8482631

[ref56] Kelly J. R. Borre Y. O' Brien C. Patterson E. el Aidy S. Deane J.. (2016). Transferring the blues: depression-associated gut microbiota induces neurobehavioural changes in the rat. J. Psychiatr. Res. 82, 109–118. doi: 10.1016/j.jpsychires.2016.07.019, PMID: 27491067

[ref57] Kenward M. G. Jones B. (2007). “15 design and analysis of cross-over trials” in Handbook of Statistics, Eds. Rao C. R. Miller J. P. Rao D. C. (Boca Raton, FL, USA: Elsevier), 464–490.

[ref58] Kim S. W. Hooker J. M. Otto N. Win K. Muench L. Shea C.. (2013). Whole-body pharmacokinetics of HDAC inhibitor drugs, butyric acid, valproic acid and 4-phenylbutyric acid measured with carbon-11 labeled analogs by PET. Nucl. Med. Biol. 40, 912–918. doi: 10.1016/j.nucmedbio.2013.06.007, PMID: 23906667 PMC3769509

[ref59] Kita A. Kohayakawa H. Kinoshita T. Ochi Y. Nakamichi K. Kurumiya S.. (2004). Antianxiety and antidepressant-like effects of AC-5216, a novel mitochondrial benzodiazepine receptor ligand. Br. J. Pharmacol. 142, 1059–1072. doi: 10.1038/sj.bjp.0705681, PMID: 15249420 PMC1575165

[ref60] Kovtun A. S. Averina O. V. Angelova I. Y. Yunes R. A. Zorkina Y. A. Morozova A. Y.. (2022). Alterations of the composition and Neurometabolic profile of human gut microbiota in major depressive disorder. Biomedicine 10:2162. doi: 10.3390/biomedicines10092162, PMID: 36140263 PMC9496097

[ref61] Lahti L Shetty SA. (2020). Tools for Microbiome Analysis in R. Available at: https://microbiome.github.io/tutorials/Betadiversity.html (Accessed October 25, 2022).

[ref62] Leneveu-Jenvrin C. Connil N. Bouffartigues E. Papadopoulos V. Feuilloley M. G. J. Chevalier S. (2014). Structure-to-function relationships of bacterial translocator protein (TSPO): a focus on Pseudomonas. Front. Microbiol. 5:631. doi: 10.3389/fmicb.2014.00631 25477872 PMC4237140

[ref63] Ludwig W. Viver T. Westram R. Francisco Gago J. Bustos-Caparros E. Knittel K.. (2021). Release LTP_12_2020, featuring a new ARB alignment and improved 16S rRNA tree for prokaryotic type strains. Syst. Appl. Microbiol. 44:126218. doi: 10.1016/j.syapm.2021.126218 34111737

[ref64] Mallick H. Rahnavard A. McIver L. J. Ma S. Zhang Y. Nguyen L. H.. (2021). Multivariable association discovery in population-scale meta-omics studies. PLoS Comput. Biol. 17:e1009442. doi: 10.1371/journal.pcbi.1009442, PMID: 34784344 PMC8714082

[ref65] Martino C. McDonald D. Cantrell K. Dilmore A. H. Vázquez-Baeza Y. Shenhav L.. (2022). Compositionally aware phylogenetic Beta-diversity measures better resolve microbiomes associated with phenotype. mSystems. 7:e0005022. doi: 10.1128/msystems.00050-22, PMID: 35477286 PMC9238373

[ref66] Mattei C. Taly A. Soualah Z. Saulais O. Henrion D. Guérineau N. C.. (2019). Involvement of the GABAA receptor α subunit in the mode of action of etifoxine. Pharmacol. Res. 145:104250. doi: 10.1016/j.phrs.2019.04.034, PMID: 31059790

[ref67] Miettinen O. S. Cook E. F. (1981). Confounding: essence and detection. Am. J. Epidemiol. 114, 593–603. doi: 10.1093/oxfordjournals.aje.a113225 7304589

[ref68] Morohaku K. Pelton S. H. Daugherty D. J. Butler W. R. Deng W. Selvaraj V. (2014). Translocator protein/peripheral benzodiazepine receptor is not required for steroid hormone biosynthesis. Endocrinology 155, 89–97. doi: 10.1210/en.2013-1556 24174323 PMC3868810

[ref69] Nearing J. T. Douglas G. M. Hayes M. G. MacDonald J. Desai D. K. Allward N.. (2022). Microbiome differential abundance methods produce different results across 38 datasets. Nat. Commun. 13:342. doi: 10.1038/s41467-022-28034-z, PMID: 35039521 PMC8763921

[ref70] Nobs S. P. Tuganbaev T. Elinav E. (2019). Microbiome diurnal rhythmicity and its impact on host physiology and disease risk. EMBO Rep. 20:e47129. doi: 10.15252/embr.201847129, PMID: 30877136 PMC6446202

[ref71] Owen D. R. J. Lewis A. J. M. Reynolds R. Rupprecht R. Eser D. Wilkins M. R.. (2011). Variation in binding affinity of the novel anxiolytic XBD173 for the 18 kDa translocator protein in human brain. Synap N Y N. 65, 257–259. doi: 10.1002/syn.20884 21132812

[ref72] Owen D. R. Phillips A. O’Connor D. Grey G. Aimola L. Nicholas R.. (2022). Human pharmacokinetics of XBD173 and etifoxine distinguish their potential for pharmacodynamic effects mediated by translocator protein. Br. J. Clin. Pharmacol. 88, 4230–4236. doi: 10.1111/bcp.15392, PMID: 35524344 PMC9545781

[ref73] Parte A. C. Sardà Carbasse J. Meier-Kolthoff J. P. Reimer L. C. Göker M. (2020). List of prokaryotic names with standing in nomenclature (LPSN) moves to the DSMZ. Int. J. Syst. Evol. Microbiol. 70, 5607–5612. doi: 10.1099/ijsem.0.004332, PMID: 32701423 PMC7723251

[ref74] Patterson A. M. Mulder I. E. Travis A. J. Lan A. Cerf-Bensussan N. Gaboriau-Routhiau V.. (2017). Human gut symbiont *Roseburia hominis* promotes and regulates innate immunity. Front. Immunol. 8:1166. doi: 10.3389/fimmu.2017.01166, PMID: 29018440 PMC5622956

[ref75] Paul S. M. Pinna G. Guidotti A. (2020). Allopregnanolone: from molecular pathophysiology to therapeutics. A historical perspective. Neurobiol Stress. 12:100215. doi: 10.1016/j.ynstr.2020.100215 32435665 PMC7231972

[ref76] Plassais J. Gbikpi-Benissan G. Figarol M. Scheperjans F. Gorochov G. Derkinderen P.. (2021). Gut microbiome alpha-diversity is not a marker of Parkinson’s disease and multiple sclerosis. Brain Commun. 3:fcab113. doi: 10.1093/braincomms/fcab113 34704023 PMC8195527

[ref77] Quillin S. J. Tran P. Prindle A. (2021). Potential roles for gamma-aminobutyric acid signaling in bacterial communities. Bioelectricity. 3, 120–125. doi: 10.1089/bioe.2021.0012, PMID: 34476387 PMC8380936

[ref78] Reagan-Shaw S. Nihal M. Ahmad N. (2008). Dose translation from animal to human studies revisited. FASEB J Off Publ Fed Am Soc Exp Biol. 22, 659–661. doi: 10.1096/fj.07-9574LSF 17942826

[ref79] Riebel M. von Pappenheim B. Kanig C. Nothdurfter C. Wetter T. C. Rupprecht R.. (2023). GABAergic effects of Etifoxine and alprazolam assessed by double pulse TMS. Pharmacopsychiatry 56, 154–161. doi: 10.1055/a-2078-4823 37220781

[ref80] Robel P. Baulieu E. E. (1985). Neuro-steroids: 3β-hydroxy-Δ5-derivatives in the rodent brain. Neurochem. Int. 7, 953–958. doi: 10.1016/0197-0186(85)90143-3 20493007

[ref81] Rochon J. (1996). Accounting for covariates observed post randomization for discrete and continuous repeated measures data. J. R. Stat. Soc. Ser. B Methodol. 58, 205–219. doi: 10.1111/j.2517-6161.1996.tb02076.x

[ref82] Rupprecht R. Wetzel C. H. Dorostkar M. Herms J. Albert N. L. Schwarzbach J.. (2022). Translocator protein (18kDa) TSPO: a new diagnostic or therapeutic target for stress-related disorders? Mol. Psychiatry 27, 2918–2926. doi: 10.1038/s41380-022-01561-3 35444254

[ref83] Sakamoto M. Sakurai N. Tanno H. Iino T. Ohkuma M. Endo A. (2022). Genome-based, phenotypic and chemotaxonomic classification of *Faecalibacterium* strains: proposal of three novel species *Faecalibacterium duncaniae* sp. nov., *Faecalibacterium hattorii* sp. nov. and *Faecalibacterium gallinarum* sp. nov. Int. J. Syst. Evol. Microbiol. 72:005379. doi: 10.1099/ijsem.0.005379 35416766

[ref84] Schlichter R. Rybalchenko V. Poisbeau P. Verleye M. Gillardin J. (2000). Modulation of GABAergic synaptic transmission by the non-benzodiazepine anxiolytic etifoxine. Neuropharmacology 39, 1523–1535. doi: 10.1016/S0028-3908(99)00253-1, PMID: 10854897

[ref85] Schloss P. D. (2018). Identifying and overcoming threats to reproducibility, replicability, robustness, and generalizability in microbiome research. mBio. 9:e00525–18. doi: 10.1128/mBio.00525-18 29871915 PMC5989067

[ref86] Seifi M. Brown J. F. Mills J. Bhandari P. Belelli D. Lambert J. J.. (2014). Molecular and functional diversity of GABA-A receptors in the enteric nervous system of the mouse colon. J. Neurosci. 34, 10361–10378. doi: 10.1523/JNEUROSCI.0441-14.2014, PMID: 25080596 PMC4115141

[ref87] Servant D. Graziani P. L. Moyse D. Parquet P. J. (1998). Treatment of adjustment disorder with anxiety: efficacy and tolerance of etifoxine in a double-blind controlled study. L’Encephale. 24, 569–574. PMID: 9949940

[ref88] Sheehan D. V. Lecrubier Y. Sheehan K. H. Amorim P. Janavs J. Weiller E.. (1998). The Mini-international neuropsychiatric interview (M.I.N.I.): the development and validation of a structured diagnostic psychiatric interview for DSM-IV and ICD-10. J. Clin. Psychiatry 59, 22–33. 9881538

[ref89] Singh N. Gurav A. Sivaprakasam S. Brady E. Padia R. Shi H.. (2014). Activation of Gpr109a, receptor for niacin and the commensal metabolite butyrate, suppresses colonic inflammation and carcinogenesis. Immunity 40, 128–139. doi: 10.1016/j.immuni.2013.12.007, PMID: 24412617 PMC4305274

[ref90] Singmann H. Kellen D. (2019). “An introduction to mixed models for experimental psychology” in New Methods in Cognitive Psychology. eds. Spieler D. Schumacher E.. 1st ed (New York: Routledge), 4–31.

[ref91] Sinha R. Abu-Ali G. Vogtmann E. Fodor A. A. Ren B. Amir A.. (2017). Assessment of variation in microbial community amplicon sequencing by the microbiome quality control (MBQC) project consortium. Nat. Biotechnol. 35, 1077–1086. doi: 10.1038/nbt.3981, PMID: 28967885 PMC5839636

[ref92] Skolnick S. D. Greig N. H. (2019). Microbes and monoamines: potential neuropsychiatric consequences of Dysbiosis. Trends Neurosci. 42, 151–163. doi: 10.1016/j.tins.2018.12.005, PMID: 30795845

[ref93] So S. Y. Savidge T. C. (2022). Gut feelings: the microbiota-gut-brain axis on steroids. Am. J. Physiol. Gastrointest. Liver Physiol. 322, G1–G20. doi: 10.1152/ajpgi.00294.2021, PMID: 34730020 PMC8698538

[ref94] Song L. Sun Q. Zheng H. Zhang Y. Wang Y. Liu S.. (2022). *Roseburia hominis* alleviates Neuroinflammation via short-chain fatty acids through histone deacetylase inhibition. Mol. Nutr. Food Res. 66:e2200164. doi: 10.1002/mnfr.202200164 35819092 PMC9787297

[ref95] Stammler F. Glasner J. Hiergeist A. Holler E. Weber D. Oefner P. J.. (2016). Adjusting microbiome profiles for differences in microbial load by spike-in bacteria. Microbiome 4:28. doi: 10.1186/s40168-016-0175-0, PMID: 27329048 PMC4915049

[ref96] Stein D. J. (2015). Etifoxine versus alprazolam for the treatment of adjustment disorder with anxiety: a randomized controlled trial. Adv. Ther. 32, 57–68. doi: 10.1007/s12325-015-0176-6, PMID: 25620535 PMC4311065

[ref97] Tan J. McKenzie C. Potamitis M. Thorburn A. N. Mackay C. R. Macia L. (2014). The role of short-chain fatty acids in health and disease. Adv. Immunol. 121, 91–119. doi: 10.1016/B978-0-12-800100-4.00003-9 24388214

[ref98] Thaiss C. A. Levy M. Korem T. Dohnalová L. Shapiro H. Jaitin D. A.. (2016). Microbiota diurnal rhythmicity programs host transcriptome oscillations. Cells 167, 1495–1510.e12. doi: 10.1016/j.cell.2016.11.003 27912059

[ref99] Thaiss C. A. Zeevi D. Levy M. Zilberman-Schapira G. Suez J. Tengeler A. C.. (2014). Transkingdom control of microbiota diurnal oscillations promotes metabolic homeostasis. Cells 159, 514–529. doi: 10.1016/j.cell.2014.09.048, PMID: 25417104

[ref100] Tindall B. J. (2014). The status of the name *Lactobacillus rogosae* Holdeman and Moore 1974. Opinion 88. Judicial Commission of the International Committee on systematics of prokaryotes. Int. J. Syst. Evol. Microbiol. 64, 3578–3579. doi: 10.1099/ijs.0.069146-0, PMID: 25288658

[ref101] Tu L. N. Zhao A. H. Stocco D. M. Selvaraj V. (2015). PK11195 effect on steroidogenesis is not mediated through the translocator protein (TSPO). Endocrinology 156, 1033–1039. doi: 10.1210/en.2014-1707, PMID: 25535830 PMC4330312

[ref102] Uhr G. T. Dohnalová L. Thaiss C. A. (2019). The dimension of time in host-microbiome interactions. mSystems. 4, e00216–e00218. doi: 10.1128/mSystems.00216-18 PMC6381226 30801030

[ref103] Valles-Colomer M. Falony G. Darzi Y. Tigchelaar E. F. Wang J. Tito R. Y.. (2019). The neuroactive potential of the human gut microbiota in quality of life and depression. Nat. Microbiol. 4, 623–632. doi: 10.1038/s41564-018-0337-x, PMID: 30718848

[ref104] Veenman L. Vainshtein A. Yasin N. Azrad M. Gavish M. (2016). Tetrapyrroles as endogenous TSPO ligands in eukaryotes and prokaryotes: comparisons with synthetic ligands. Int. J. Mol. Sci. 17:880. doi: 10.3390/ijms17060880, PMID: 27271616 PMC4926414

[ref105] Verhaar B. J. H. Hendriksen H. M. A. de Leeuw F. A. Doorduijn A. S. van Leeuwenstijn M. Teunissen C. E.. (2021). Gut microbiota composition is related to AD pathology. Front. Immunol. 12:794519. doi: 10.3389/fimmu.2021.794519 35173707 PMC8843078

[ref106] Verleye M. Akwa Y. Liere P. Ladurelle N. Pianos A. Eychenne B.. (2005). The anxiolytic etifoxine activates the peripheral benzodiazepine receptor and increases the neurosteroid levels in rat brain. Pharmacol. Biochem. Behav. 82, 712–720. doi: 10.1016/j.pbb.2005.11.013, PMID: 16388839

[ref107] Verster J. C. Volkerts E. R. (2004). Clinical pharmacology, clinical efficacy, and behavioral toxicity of alprazolam: a review of the literature. CNS Drug Rev. 10, 45–76. doi: 10.1111/j.1527-3458.2004.tb00003.x, PMID: 14978513 PMC6741717

[ref108] Wagner B. D. Grunwald G. K. Zerbe G. O. Mikulich-Gilbertson S. K. Robertson C. E. Zemanick E. T.. (2018). On the use of diversity measures in longitudinal sequencing studies of microbial communities. Front. Microbiol. 9:1037. doi: 10.3389/fmicb.2018.01037, PMID: 29872428 PMC5972327

[ref109] Weiss S. Xu Z. Z. Peddada S. Amir A. Bittinger K. Gonzalez A.. (2017). Normalization and microbial differential abundance strategies depend upon data characteristics. Microbiome. 5:27. doi: 10.1186/s40168-017-0237-y, PMID: 28253908 PMC5335496

[ref110] Williams E. J. (1949). Experimental designs balanced for the estimation of residual effects of treatments. Aust J Sci Res Ser Phys Sci Aust J Chem. 2:149. doi: 10.1071/CH9490149

[ref111] Yeliseev A. A. Krueger K. E. Kaplan S. (1997). A mammalian mitochondrial drug receptor functions as a bacterial “oxygen” sensor. Proc. Natl. Acad. Sci. U. S. A. 94, 5101–5106. doi: 10.1073/pnas.94.10.5101, PMID: 9144197 PMC24638

[ref112] Yurdaydin C. Walsh T. J. Engler H. D. Ha J. H. Li Y. Jones E. A.. (1995). Gut bacteria provide precursors of benzodiazepine receptor ligands in a rat model of hepatic encephalopathy. Brain Res. 679, 42–48. doi: 10.1016/0006-8993(95)00241-H, PMID: 7648264

[ref113] Zheng P. Zeng B. Zhou C. Liu M. Fang Z. Xu X.. (2016). Gut microbiome remodeling induces depressive-like behaviors through a pathway mediated by the host’s metabolism. Mol. Psychiatry 21, 786–796. doi: 10.1038/mp.2016.44, PMID: 27067014

[ref114] Zhou H. He K. Chen J. Zhang X. (2022). LinDA: linear models for differential abundance analysis of microbiome compositional data. Genome Biol. 23:95. doi: 10.1186/s13059-022-02655-5, PMID: 35421994 PMC9012043

[ref115] Zmora N. Zilberman-Schapira G. Suez J. Mor U. Dori-Bachash M. Bashiardes S.. (2018). Personalized gut mucosal colonization resistance to empiric probiotics is associated with unique host and microbiome features. Cells 174, 1388–1405.e21. doi: 10.1016/j.cell.2018.08.041, PMID: 30193112

