# Supplementary material for: Short-term effects of etifoxine on human gut microbiome in healthy men
Source: Front Neurosci. 2023 Nov 14;17:1188847. doi: 10.3389/fnins.2023.1188847 (PMC10704910; doi:10.3389/fnins.2023.1188847)
Supplement: Supplementary file 1 [file Data_Sheet_1.pdf]

Supplementary Material

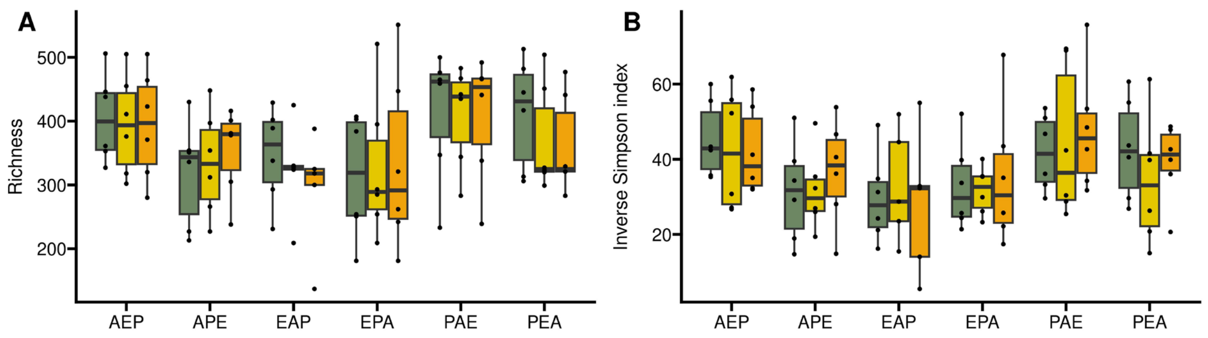

Figure S1: Alpha diversity of gut microbiome grouped in sequences over treatment periods across complete trial. Name of sequence groups corresponds to first letters of treatments (*A* = alprazolam, *E* = etifoxine, *P* = placebo). Treatment periods are colored in *green* (T1), *yellow* (T2) and *orange* (T3). Two different indices for alpha diversity are shown to account for microbial richness alone (panel A) and proportional abundance (*Inverse Simpson*, panel B).

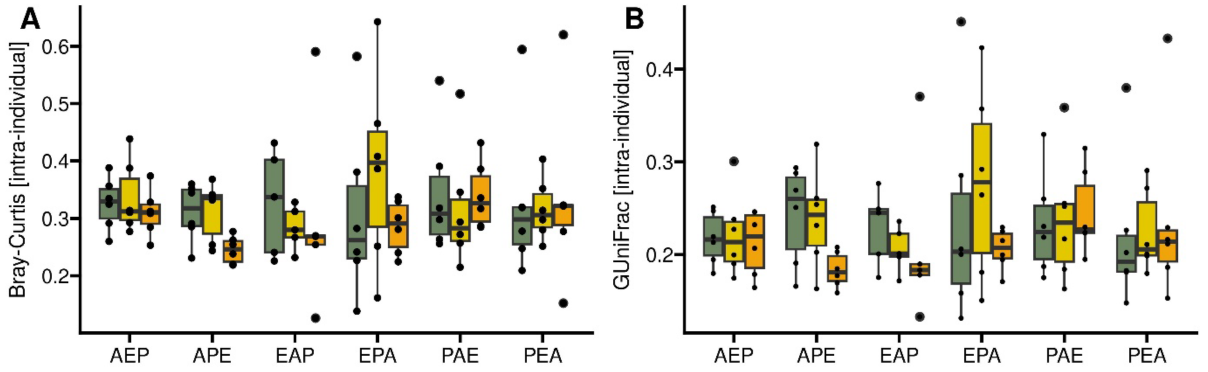

Figure S2: Intra-individual beta diversity of gut microbiome grouped in sequences over treatment periods across complete trial. Name of sequences corresponds to first letters of treatments (*A* = alprazolam, *E* = etifoxine, *P* = placebo). Treatment periods are colored in *green* (T1), *yellow* (T2) and *orange* (T3). Two different indices for beta diversity are shown. *Bray-Curtis* (panel A) accounts for dissimilarities on zOTU-levels and weighs these according to their relative abundances. *Generalized UniFrac* (panel B) additionally incorporates phylogenetic distances originating from genetic sequence dissimilarities. Generalized UniFrac with alpha=1.0 corresponds to a conventional weighted UniFrac representation. Here, keeping the default of alpha=0.5 reduces the weights of highly abundant species.

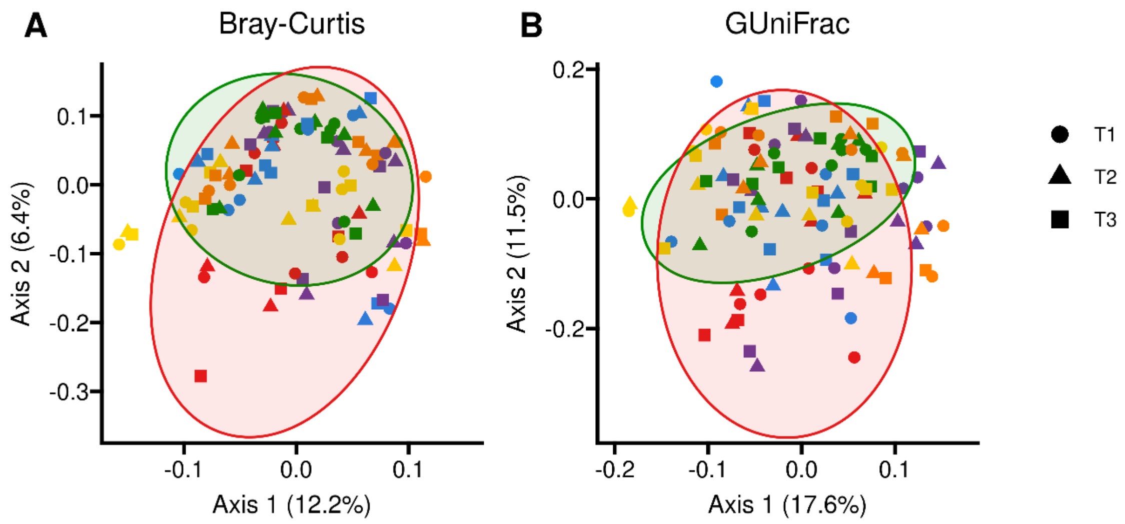

Figure S3: Beta diversity of gut microbiome among all samples across complete trial marked by their sequences (*symbol colors*) and treatment periods (*symbol shapes*). Sequences AEP and EAP are separately circumscribed with ellipses in the color of these sequence groups. Beta dispersion of Bray-Curtis distance matrices was significantly different in these two groups. Two different indices for beta diversity are shown. *Bray-Curtis* (panel A) accounts for dissimilarities on zOTU-levels and weighs these according to their relative abundances. *Generalized UniFrac* (panel B) additionally incorporates phylogenetic distances originating from genetic sequence dissimilarities. Generalized UniFrac with alpha=1.0 corresponds to a conventional weighted UniFrac representation. Here, keeping the default of alpha=0.5 reduces the weights of highly abundant species. Same data as in Figure 5. Color code for sequences is identical to Figure S4.

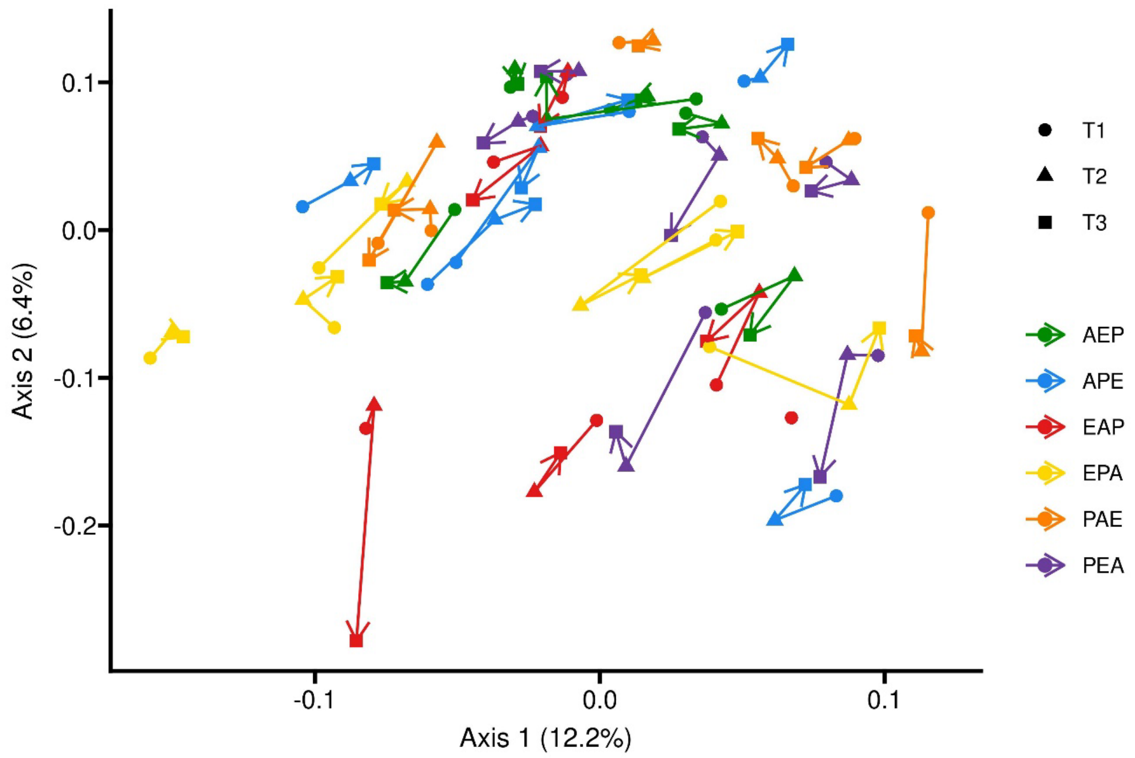

Figure S4: Beta diversity of gut microbiome among all samples across complete trial marked by their sequences (*colors*), treatment periods (*symbol shapes*) and time-series of individual participants (*arrows*). Beta diversity is shown based on Bray-Curtis distances. This index accounts for dissimilarities on zOTU-levels and weighs these according to their relative abundances. Same data as in Figure 5, panel A and as in Figure S3, panel A.

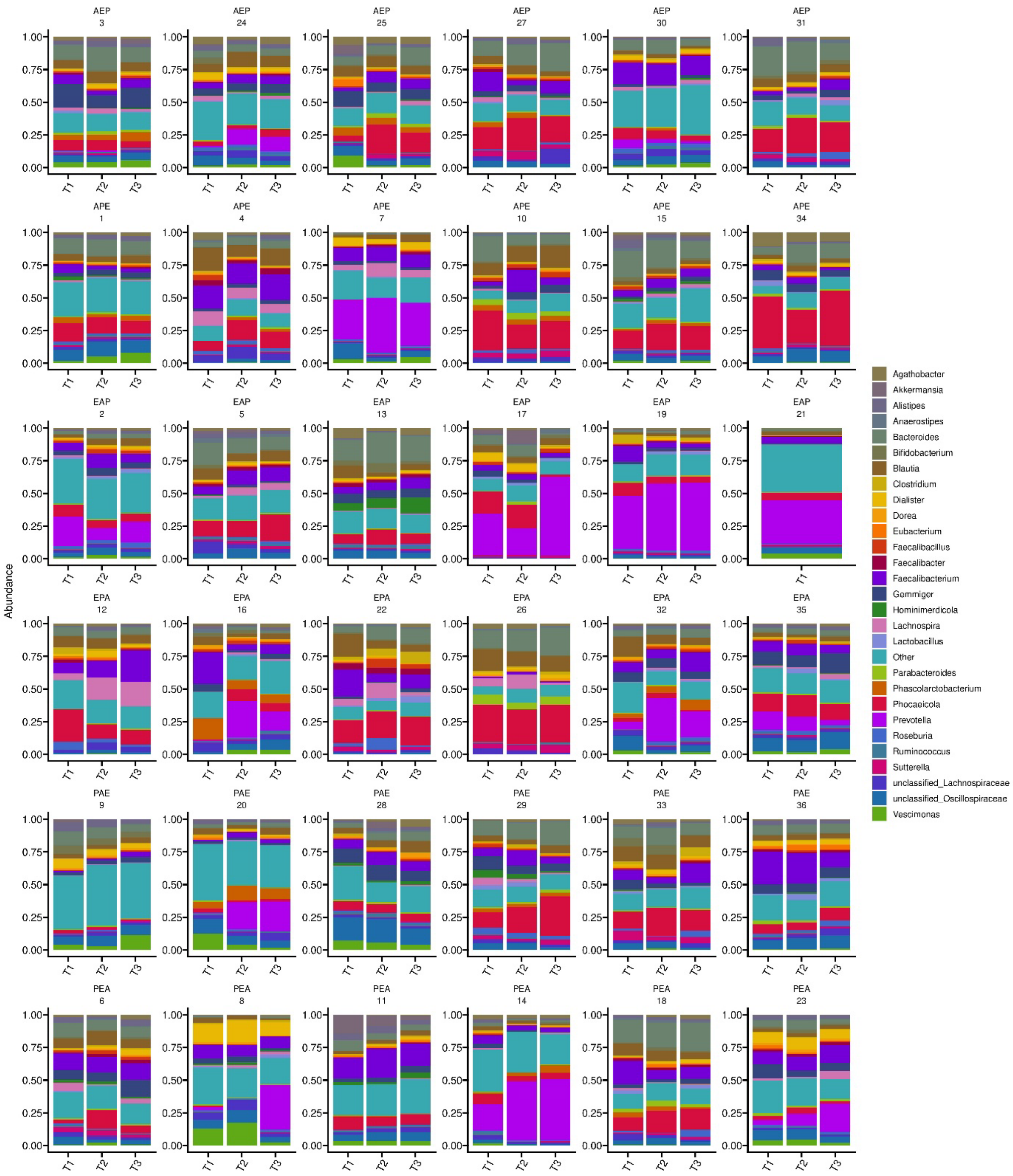

Figure S5: Relative abundance taxonomy barplots on genus level for each participant of a sequence (ordered in rows) over periods (i.e. time-points T1, T2 and T3). zOTUs with a sequence count above 100 are included in this representation. Top 30 most abundant genera are shown individually as described in the legend box to the right. Relative abundance data averaged by treatment for all participants shown, here, is presented in Figure 7.

Mean relative abundance [%]	Frequency [%]	Phylum	Class	Order	Family	Genus
11.4	100.0	Bacteroidota	Bacteroidia	Bacteroidales	Bacteroidaceae	Phocaeicola
10.2	100.0	Bacteroidota	Clostridia	Eubacteriales	Oscillospiraceae	Faecalibacterium
7.5	100.0	Bacteroidota	Bacteroidia	Bacteroidales	Bacteroidales (unclassified)	unidentified
7.4	100.0	Bacteroidota	Bacteroidia	Bacteroidales	Bacteroidaceae	Bacteroides
7.0	69.4	Bacteroidota	Bacteroidia	Bacteroidales	Prevotellaceae	Prevotella
5.5	100.0	Bacillota	Clostridia	Eubacteriales	Oscillospiraceae	Oscillospiraceae (unclassified)
5.1	94.4	Bacillota	Clostridia	Eubacteriales	Oscillospiraceae	Gemmiger
4.9	100.0	Bacillota	Clostridia	Eubacteriales	Lachnospiraceae	Blautia
3.0	100.0	Bacillota	Clostridia	Eubacteriales	Lachnospiraceae	Lachnospira
2.3	100.0	Bacillota	Clostridia	Eubacteriales	Lachnospiraceae	Lachnospiraceae (unclassified)

Table S1: Relative abundance of the ten most abundant gut microbial genera from the bacterial domain as measured at baseline
